# A new role of hindbrain boundaries as pools of neural stem/progenitor cells regulated by Sox2

**DOI:** 10.1186/s12915-016-0277-y

**Published:** 2016-07-08

**Authors:** Yuval Peretz, Noa Eren, Ayelet Kohl, Gideon Hen, Karina Yaniv, Karen Weisinger, Yuval Cinnamon, Dalit Sela-Donenfeld

**Affiliations:** Koret School of Veterinary Medicine, The Robert H. Smith Faculty of Agriculture, Food and Environment, The Hebrew University of Jerusalem, Rehovot, 76100 Israel; Department of Biological Regulation, Weizmann Institute of Science, Rehovot, Israel; Department of Stem Cell and Regenerative Biology, Harvard Stem Cell Institute, Harvard University, Cambridge, MA USA; Institute of Animal Sciences, Department of Poultry and Aquaculture Sciences, Agricultural Research Organization, The Volcani Center, Bet Dagan, Israel

**Keywords:** Hindbrain boundaries, Rhombomere, Sox2, Neural differentiation, Neural stem/progenitors

## Abstract

**Background:**

Compartment boundaries are an essential developmental mechanism throughout evolution, designated to act as organizing centers and to regulate and localize differently fated cells. The hindbrain serves as a fascinating example for this phenomenon as its early development is devoted to the formation of repetitive rhombomeres and their well-defined boundaries in all vertebrates. Yet, the actual role of hindbrain boundaries remains unresolved, especially in amniotes.

**Results:**

Here, we report that hindbrain boundaries in the chick embryo consist of a subset of cells expressing the key neural stem cell (NSC) gene Sox2. These cells co-express other neural progenitor markers such as Transitin (the avian Nestin), GFAP, Pax6 and chondroitin sulfate proteoglycan. The majority of the Sox2^+^ cells that reside within the boundary core are slow-dividing, whereas nearer to and within rhombomeres Sox2^+^ cells are largely proliferating. In vivo analyses and cell tracing experiments revealed the contribution of boundary Sox2^+^ cells to neurons in a ventricular-to-mantle manner within the boundaries, as well as their lateral contribution to proliferating Sox2^+^ cells in rhombomeres. The generation of boundary-derived neurospheres from hindbrain cultures confirmed the typical NSC behavior of boundary cells as a multipotent and self-renewing Sox2^+^ cell population. Inhibition of Sox2 in boundaries led to enhanced and aberrant neural differentiation together with inhibition in cell-proliferation, whereas Sox2 mis-expression attenuated neurogenesis, confirming its significant function in hindbrain neuronal organization.

**Conclusions:**

Data obtained in this study deciphers a novel role of hindbrain boundaries as repetitive pools of neural stem/progenitor cells, which provide proliferating progenitors and differentiating neurons in a Sox2-dependent regulation.

**Electronic supplementary material:**

The online version of this article (doi:10.1186/s12915-016-0277-y) contains supplementary material, which is available to authorized users.

## Background

During animal development, groups of cells with similar fates and functions are often separated from other cells by the formation of sharp boundaries. Such boundaries are fundamental during the development of the central nervous system (CNS), where they act as organizing centers to pattern the tissue and localize differently-fated cells via the secretion of signaling molecules [1, 2]. The hindbrain serves as an excellent system to study regional specification and pattern formation, as its early development is devoted to the formation of 7 to 8 repetitive segments, termed rhombomeres, along the anterior-posterior (AP) axis of all vertebrates [3]. Each rhombomere is a lineage-restricted compartment which underlies unique patterns of gene expression, neural crest migration and neuronal differentiation [4–12]. Individual rhombomeres are separated from their neighbors by well-defined boundaries. Contrary to rhombomeres, hindbrain boundaries (HBs) share the same molecular and cellular characteristics along the hindbrain, such as a unique fan-shaped morphology, enriched extracellular matrix (ECM), slow proliferation rate, and reduced interkinetic nuclear cell migration [5, 7, 13–19]. Although HBs were identified decades ago, their role during hindbrain development remains largely unknown, especially in amniotes.

The midbrain-hindbrain boundary (MHB) is a well-defined domain located at the border between the midbrain and rhombomere 1. The MHB acts as an organizing center that expresses signaling factors, such as Wnts and FGF8, and regulates distinct gene expression patterns and neuronal fates of midbrain and anterior hindbrain cells [20–24]. Numerous studies have shown that cells within the MHB remain as slowly proliferating, non-differentiating progenitor cell populations [25–27]. Studies in zebrafish and mice have highlighted the role of the Notch effector group of *Hes* genes, which are expressed in MHB cells, to repress them from undergoing differentiation while promoting neurogenesis in the adjacent domains [28–33].

Do HBs also act as signaling centres to organize hindbrain development? Similar to the MHB, HB cells (HBCs) express a variety of signaling molecules, including FGFs (in mice and chicks) or Wnts (in zebrafish) [20, 34–39]. Additionally, repressors of neural differentiations, such as Hes1, Id1 and Radical Fringe, were reported to be expressed in HBCs of chick, mice or fish [40–42]. We have previously found that HBs of chick embryos are controlling the downregulation of different genes initially expressed within rhombomeres (FGFs, Pax6, follistatin) [43]. Moreover, recent zebrafish studies have shown how HBs, which express the guidance cue semaphorin, drive the clustering of neurons away from the boundaries to the center of rhombomeres [44]. All these data support the possibility that HBs are involved in gene expression patterns and neural localization in different species. Yet, whether HBs are indeed organizing centres that regulate neural differentiation in the hindbrain is not clear.

SRY-related HMG-box 2 gene (Sox2), a member of the SoxB transcription factor family [45–47], is a fundamental factor in self-renewal and multipotency of embryonic and adult neural stem cells (NSCs). It plays key roles during CNS development, such as in survival, proliferation and maintenance of NSCs [48–50], as well as in the acquisition of neural/glial identity [51–61]. As expected from the key role of Sox2 in neural progenitor cells (NPCs), previous studies have shown that early in neural tube development, Sox2 is expressed along the entire hindbrain [62, 63]. Here, we present that, at later stages of development (St.18 chick embryos), Sox2 becomes localized to HBs, along with multiple other classical NPC markers. Furthermore, we demonstrate that the Sox2-expressing HBCs contribute proliferating cells to adjacent rhombomeres, and also directly differentiate into Sox2-negative neurons at the boundaries. The significant role Sox2 plays in mediating hindbrain neural differentiation and cell division patterning is shown by loss- and gain-of-function assays in vivo and in vitro. Overall, our data highlight a novel role for HBs as repetitive pools of NPCs that coordinate neural differentiation in the developing hindbrain.

## Results

### Sox2 converges from the entire hindbrain to its boundaries with time

**Fig. 1:**
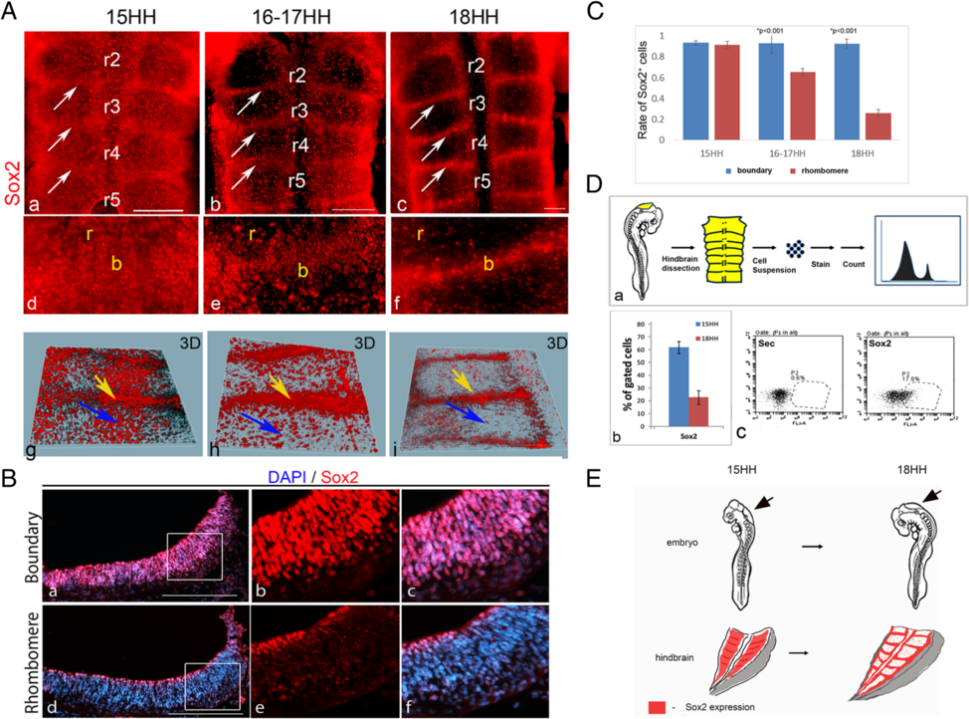
Expression of Sox2 in chick hindbrain boundaries. **A** Representative flat-mounted views of hindbrains of 15HH (**a**,**d**,**g**), 16-17HH (**b**,**e**,**h**) and 18HH (**c**,**f**,**i**) chick embryos immunostained for Sox2 (n = 10 for each group). White arrows indicate hindbrain boundaries (HBs). Higher magnification of r3/4 boundary areas, respectively (**d**–**f**). Views of confocal-generated 3D models, with yellow arrows indicating HBs and blue arrows indicating rhombomeres (**g**–**i**). **B** Representative transverse section of a boundary (**a**–**c**) and rhombomere (**d**–**f**) region from 18HH hindbrain, stained with Sox2 and DAPI (n = 5). **C** Quantification of Sox2^+^ cells in rhombomeres vs*.* boundaries in 15HH, 16-17HH and 18HH hindbrains (n = 5 embryos for each stage). *P* value obtained by using *t* test. **D** Protocol for marker quantification shows dissociation of the hindbrain (15/18HH) into single cells, immunostaining and analysis by flow cytometry (**a**). Quantification of Sox2^+^ cells as percentage out of total gated cells in 15HH and 18HH hindbrains (**b**). Representative flow cytometry plots for this experiment (**c**). **E** Illustration of the confinement of Sox2^+^ cells to HBs during maturation from 15HH to 18HH. Arrows indicate the hindbrain in the whole embryo. r = rhombomere, b = boundary, Sec = secondary Ab. only, prop = proportion. Scale bars = 100 μm

**Fig. 2:**
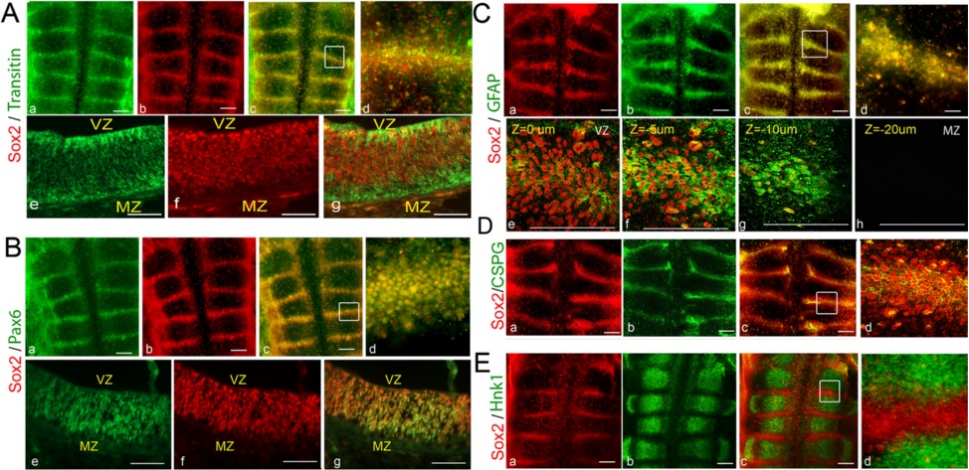
Co-expression of Sox2 with neural stem cells and cell-surface markers in the hindbrain. Representative flat-mount or section views of 18HH chick hindbrains that were co-stained for Sox2 and various cellular markers (n = 10 for each marker). **A** Hindbrain co-stained for Sox2 and Transitin. Flat-mounted views of single (**a**,**b**) or merged (**c**,**d**) channels. Higher magnification view of boundary area marked in (**c**) is presented in (**d**). Transverse section of single (**e**,**f**) or merged (**g**) channels of a boundary area. **B** Hindbrain co-stained for Sox2 and Pax6. Flat-mounted views of single (**a**,**b**) or merged (**c**,**d**) channels. Higher magnification view of boundary area marked in (**c**) is presented in (**d**). Transverse section of single (**e**,**f**) or merged (**g**) channels of a boundary area. **C** Hindbrain co-stained for Sox2 and GFAP. Flat-mounted views of single (**a**,**b**) or merged (**c**,**d**) channels. Higher magnification view of boundary area marked in (**c**) is presented in (**d**). Confocal Z-stack images of a boundary area (**e**–**h**). **D** Hindbrain co-stained for Sox2 and CSPG. Flat-mounted views of single (**a**,**b**) or merged (**c**,**d**) channels. Higher magnification view of boundary area marked in (**c**) is presented in (**d**). **E** Hindbrain co-stained for Sox2 and Hnk1. Flat-mounted views of single (**a**,**b**) or merged (**c**,**d**) channels. Higher magnification view of area marked in (**c**) is presented in (**d**). VZ = ventricular zone, MZ = mantle zone. Scale bars = 100 μm

**Fig. 3:**
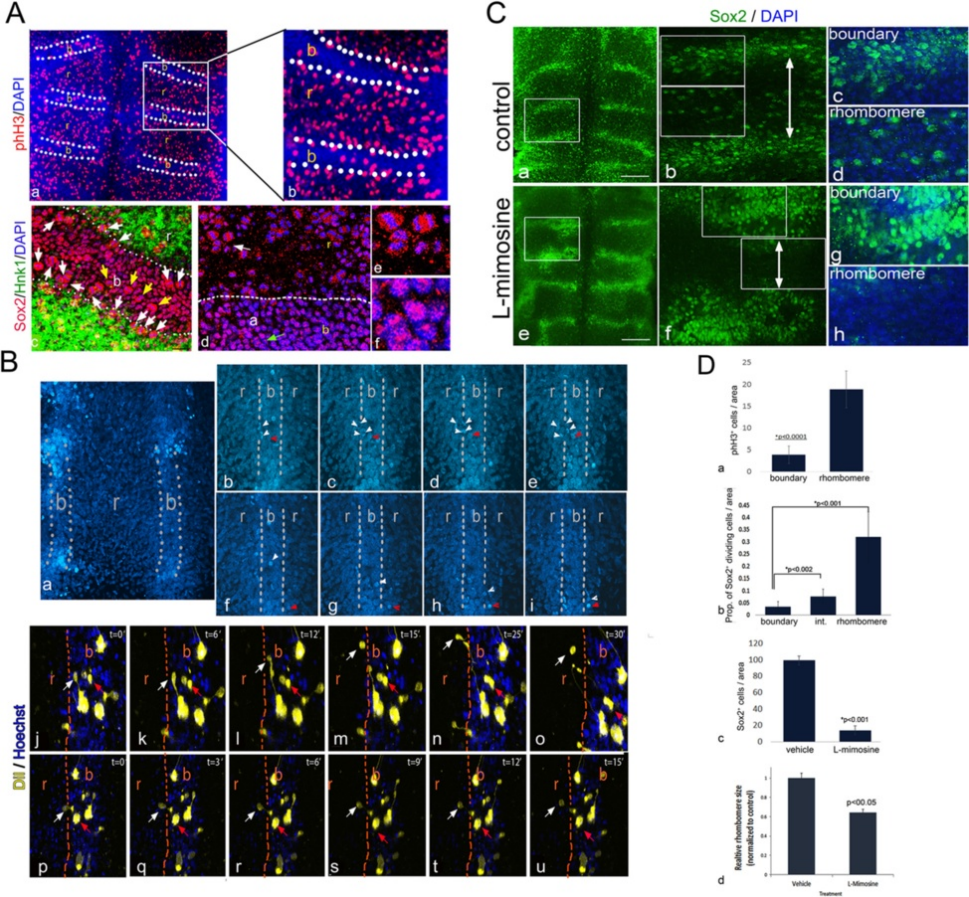
The proliferation and migration state of Sox2^+^ cells in hindbrain boundaries. **A** Representative flat-mount of 18HH hindbrain stained for phH3 and DAPI (**a**,**b**). Arrows indicate boundary domains with fewer phH3^+^ cells. Enlargement of boxed area presented in (**b**) (n = 10). Flat-mounted view of r4/5 boundary of 18HH hindbrain stained for Sox2, Hnk1 and DAPI (**c**). White Arrows indicate dividing cells at the lateral edges of the boundary. Yellow arrow indicate mitotic Sox2+ cells at boundary core. Flat-mounted view of r4/5 boundary of 18HH hindbrain stained for Sox2 and DAPI. High magnification of rhombomere area (**e**), indicated by white arrow in (**d**), shows Sox2^+^ dividing cells; high magnification of boundary core (**f**), indicated by green arrow in (**d**), shows Sox2 in non-mitotic cells. Dashed line in (**b**,**d**) indicates boundary–rhombomere intersection, based on phH3 and Hnk1 staining. **B** Representative time-lapse microscopy of boundary cells labeled with Hoechst (n = 10) (**a**–**u**) and CM-DiI (n = 8) (**j**–**u**). Low magnification view showing r4 and its adjacent boundaries (**a**). Higher magnification views of a boundary and a rhombomere (**b**–**u**). White arrowheads/arrows indicate dividing and migrating boundary cells that are contributed to the adjacent rhombomere. Red arrows/arrowheads indicate cells that remain still in the boundary (**b**–**e**, **j**–**u**) or rhombomere (**f**–**i**) throughout the experiment. Dashed line indicates boundary–rhombomere intersection. **C** Representative flat-mounted hindbrains treated with vehicle (DMEM:F12; **a**–**d**) or L-mimosine (**e**–**h**) and stained for Sox2 and DAPI (n = 14 in each group). Magnification of boxed areas in (**a**,**e**) are shown in (**b**,**f**). Magnifications of boundary and rhombomere areas from (**b**,**f**) are shown in (**c**,**d**,**g**,**h**, respectively). **D** Quantification of phH3^*+*^ cells per area in boundary vs. rhombomere (**a**). Quantification of the proportion of dividing Sox2^+^ cells in boundary, rhombomere and intersection areas (**b**). Quantification of Sox2^+^ cells per area in L-mimosine-treated hindbrains vs*.* controls (**c**). Quantification of r3 and r4 sizes in control and L-mimosine-treated hindbrains (**d**). In all analyses, *P* values were obtained using the *t* test. r = rhombomere, b = boundary, Prop = proportion. Scale bars in A,C = 100 μm; in B = 50 μm

Boundaries of the developing hindbrain become morphologically distinct soon after rhombomere formation [64]. Yet, in terms of marker expression, boundaries fully adopt their identity much later, around stage 17 [15, 16, 34, 43, 65, 66]. Individual rhombomeres express specific markers and adopt unique differentiation fates [7, 67]. The facts that boundary-specific genes are shared by all boundaries and that rhombomere markers (i.e., Hoxb1, Krox20) are lost from boundary cells over time [15] led us to hypothesize that boundaries may differ from rhombomeres also in their neural differentiation state. To test this hypothesis, we used the chick embryo and performed immunostaining for Sox2, a master regulator of neural development that is expressed in NPCs and gets downregulated upon differentiation [49, 68–70]. Previous studies have shown that Sox2 is broadly expressed in the early chick hindbrain [52, 71]. Here, we examined Sox2 expression at later stages (st.15–18), when multiple other boundary markers are fully expressed [34, 72]. Determination of the boundary regions was made based on the clear morphological bulges at these sites which contain an accumulation of cell bodies at the ventricular side compared to rhombomeres, as shown by DAPI nuclear staining, as well as by the specialized expression of different markers at these regions (Fig. 1A, Figs. 2 and 3; Additional file 1) [15, 34, 43, 72, 73]. Notably, DAPI-negative gaps are present in the mantle-most layer of boundaries, which reflect nuclei-free domains (Additional file 1), consistent with previously published data [14–16]. At st.15, Sox2 expression is detected in both rhombomeres and boundaries (Fig. 1Aa,d; n = 10). At st.16–17, Sox2^+^ cells are still present in rhombomeres, yet enhanced Sox2 expression can be detected in HBs (Fig. 1Ab,e; n = 10). At st.18, Sox2 expression is largely localized at the boundaries, with some Sox2^+^ cells still found scattered within rhombomeres (Fig. 1Ac,f; n = 10). A 3-dimensional (3D) model constructed from 30-μm thick hindbrain tissue further confirmed the tendency of Sox2^+^ cells to concentrate at the boundaries over time (Fig. 1Ag–i). The enrichment of Sox2 expression in st.18 HBs was also confirmed in transverse sections, where enhancement of Sox2^+^ cells could be identified in sections originating from boundaries, compared to fewer Sox2 cells in rhombomere-derived sections (Fig. 1Ba–f; n = 5). Noticeably, the data obtained from the sections (Fig. 1B) and the confocal analysis of whole hindbrain cells (Additional file 1) shows no larger amount of cells in the boundaries compared to rhombomeres [14], ruling out the option that the enriched Sox2 expression at HBs of st.18 embryos is due to a general increase in cell density at these sites.

To quantify the changes in Sox2 expression from st.15–18, Sox2^+^ cells were counted in eight comparable areas taken from boundaries of rhombomeres 3/4 and 4/5, or from rhombomeres 4,5 (Fig. 1C; n = 5 embryos for each stage). The number of Sox2^+^ cells was normalized to the same total number of DAPI^+^ nuclei in each compartment. Hindbrains of st.15 showed similar numbers of Sox2-expressing cells (~90 %) in boundaries and rhombomeres. Yet, at later stages, Sox2 expression became significantly lower in the rhombomeres (~62 % and 22 % in st.16–17 and 18, respectively), whereas HBs remained with approximately 90 % of cells expressing Sox2. This result further demonstrates a gradual reduction in Sox2^+^ cells in rhombomeres together with sustained Sox2 expression at HBs. The relative abundance of total Sox2^+^ cells within the hindbrain of the different stages was also measured using flow cytometry. Cell suspensions were prepared from freshly dissected hindbrains and immunostained for Sox2 (Fig. 1Da). A 3-fold reduction in the number of Sox2^+^ cells was evident in st.18 compared to st.15 hindbrains, from approximately 60 % to approximately 20 % (Fig. 1Db, representative plot of flow cytometry analysis presented in Fig. 1Dc). Overall, the general decrease in Sox2-expressing cells that is observed in the hindbrains of st.15–18 embryos, together with the continual Sox2 expression in boundaries (Fig. 1E), is in agreement with other NPC domains that shrink from broader areas to well-defined niches during CNS development [74–76].

### Sox2 is expressed with other neural progenitor markers in HBs

As Sox2 is a landmark of NPCs, we set out to examine whether other progenitor markers co-localize with Sox2 in HBs. The expression of the intermediate filament Transitin (the avian homologue of Nestin), the transcription factor Pax6, and glial fibrillary acidic protein (GFAP) was tested at st.18. All these proteins are known to be co-expressed with Sox2 in different NSCs [48–50, 61, 77–80]. Notably, Pax6 was previously found to be enriched at chick HBs [15, 72]. Immunostaining of embryos revealed the co-localization of these markers with Sox2 within HBs (Fig. 2A–C; n = 10 for each marker). Transverse sections (Fig. 2A,B) or Z-stack confocal images (Fig. 2C) of boundary regions further confirmed this finding. This analysis also showed the expression of Sox2, GFAP and Pax6 at the ventricular and sub-ventricular boundary domains excluding the mantle zone, as expected from progenitor cells upon their migration and differentiation (Fig. 2Be–g, Ce–h). At variance, while Transitin and Sox2 are also co-expressed, Transitin expression extended to neurofilaments in the pial/Sox2^–^ domains of HBs (Fig. 2Ae–g), in agreement with previous studies [81, 82]. Quantification of Transitin expression by flow cytometry confirmed our observation, showing approximately 40 % of hindbrain cells expressing this marker (Additional file 2Aa–c). Together, these results support the hypothesis of the enriched presence of typical NPCs at ventricular and sub-ventricular layers of HBs.

As Sox2 expression is decreased in the total hindbrain and remains in the boundaries between st.15–18 (Fig. 1), we next examined whether the other NPC markers display similar dynamics. Analysis of Transitin and Pax6 showed that both are broadly expressed at st.15, together with Sox2 (Additional file 2B). This result supports the global reduction of NPCs in the whole hindbrain with time and their retention in its boundaries.

Chondroitin sulfate proteoglycan (CSPG) is an ECM molecule previously shown to be enriched in chick HBs [15, 34, 43]. Interestingly, several studies demonstrated the expression of CSPG in NSCs and suggested a role for this proteoglycan in the maintenance of NSC niches [83, 84]. Analysis of st.18 hindbrains revealed co-localization between Sox2 and CSPG at HBs, such that each Sox2^+^ cell at the boundary core is surrounded by CSPG (Fig. 2D; n = 10). Quantification of stained hindbrain cells by flow cytometry showed approximately 10 % of the hindbrain cells to be CSPG^+^ (Additional file 2Ad–f).

Finally, to fully demonstrate the restricted localization of putative NPCs between rhombomeres, we searched for a general marker which is expressed in all hindbrain rhombomeres at st.18, but is excluded from HBs. Surprisingly, the glycan epitope HNK1 (human CD57, largely used as a neural crest cell marker) [85], was found to be such a pan-rhombomeric marker (Fig. 2Eb). Co-labeling of Sox2 and HNK1 showed a clear segregation between rhombomere and boundary domains (Fig. 2Ea–d; n = 12). Altogether, these results support the localization of Sox2^+^ progenitorial cell populations with their enriched ECM at HBs.

### The proliferation state of Sox2-expressing cells in HBs

NSC/NPCs have been demonstrated as slow-proliferating/quiescent cell populations in different CNS domains, such as in the telencephalon and retina [86, 87]. These slow-dividing NPCs often exhibit radial glia properties and give rise to transiently amplifying progenitors, which in turn give rise to neuronal precursors that further differentiate and migrate [88]. Notably, studies from the Lumsden group have revealed that HBCs display slower cell divisions compared to rhombomeric cells [14]. This supports our hypothesis regarding the presence of putative NPCs in HBs. To further test this possibility, st.18 hindbrains were stained for phosphorylated histone H3 (phH3), which labels cells at the M phase of the cell cycle [89]. Cells labeled with phH3 were broadly expressed within rhombomeres and significantly reduced in HBs (Fig. 3Aa,b). Counting phH3^+^ cells in 20 comparable areas of boundary and rhombomeres from seven embryos confirmed a ratio of approximately 4:1 mitotic cells in the rhombomeres versus boundaries (Fig. 3Da). Hindbrains were also stained for Sox2 and HNK1 to demarcate boundary-rhombomere interfaces, as well as with DAPI to visualize mitotic divisions. This staining validated the enrichment of non-dividing Sox2^+^ cells at the center of HBs (Fig. 3Ac). Yet, closer to the interface with rhombomere/HNK1^+^ domains, more dividing Sox2^+^ cells could be found (Fig. 3Ac). Staining with Sox2/DAPI alone further confirmed the enrichment of non-dividing Sox2^+^ cells at the center of the HBs, compared to the finding of dividing Sox2^+^ cells closer to and within rhombomeres (Fig. 3Ad–f, n = 10). Quantification of this data clearly demonstrated the low levels of Sox2^+^ dividing cells in the boundary and the elevation in their number during the transition from the boundary to rhombomere (Fig. 3Db, n = 6). Altogether, these data indicate that the majority of Sox2^+^ cells that constitute the boundaries are slow-dividing, whereas most Sox2^+^ cells in rhombomeres are dividing. Moreover, some boundary Sox2^+^ cells which are located nearer the rhombomere do divide, suggesting an increase in Sox2^+^ cell division from the boundary core to its edges, where they meet the rhombomeres.

The cell division pattern within HBs raised the possibility that the slow-dividing Sox2^+^ NPCs give rise to faster-dividing Sox2^+^ progenitors that contribute Sox2^+^ cells to adjacent rhombomeres. To examine this, st.18 hindbrains were incubated with Hoechst to stain the nuclei of living cells, and cell movements were analyzed for 6–8 h using time lapse confocal microscopy (Fig. 3Ba–i; n = 8, see movie in Additional file 3). Observation of the r4/5 boundary revealed an ongoing directional contribution of cells from the boundary to the adjacent rhombomere by enhanced cell divisions at the boundary-rhombomere interface (Fig. 3Bb–e, white arrowhead), as well as by cell migration (Fig. 3Bf–i, white arrowhead). Additional cells did not move during this time window in the boundary of the rhombomere either (Fig. 3Ba–i, red arrowheads). In addition, we labeled a small number of st.18 boundary cells with CM-DiI and analyzed their movement in time lapse for 2–8 h (Fig. 3Bj–u, n = 10, see movies in Additional files 4 and 5). CM-DiI is a fluorescent lipophilic dye useful for staining the membranes of specific cells. This kind of labeling allows tracking of the behavior and descendants of labelled cells. Similar to the Hoechst-stained hindbrains, some DiI-labeled cells within the boundary could be found migrating to the adjacent rhombomere, whereas others were captured during their division and migration of one daughter cell toward the rhombomere (Fig. 3Bj–u, white arrows). Additional cells remained still during this time frame (Fig. 3Bj–u, red arrows). Notably, some of the migrating cells were linked to boundary cells. This phenomenon may suggest the presence of intercellular bridges that are formed by daughter cells as they move apart and migrate after mitosis [90]. Furthermore, we also performed DiI labeling of larger areas of boundaries versus rhombomeres and analyzed the tissue on the next day (Additional file 6, n = 5). A directional expansion of DiI-labelled cells was demonstrated from the boundary to the rhombomere. Conversely, DiI expansion in the rhombomere was more radial and stained cells were found in all directions. Collectively, these results demonstrated that the boundaries contribute cells to adjacent rhombomeres by cell migration and division.

If indeed HBs provide proliferating Sox2^+^ cells to rhombomeres, arrest of cell division may lead to accumulation of Sox2^+^ cells at the boundaries without their movement to the rhombomeres. To examine this possibility, st.18 embryos were treated for 6 h with L-mimosine and stained for Sox2. This substance arrests the cell cycle at late G1 phase and has been shown to efficiently block cell division in chick embryos [91, 92]. Control embryos (n = 14) retained the typical pattern of enhanced Sox2 staining in HBs and fewer Sox2^+^ cells at rhombomeres (Fig. 3Ca–d, as also shown in Fig. 1A). Treatment with L-mimosine (n = 14) led to a dramatic thickening of the Sox2^+^ domains between rhombomeres together with a marked reduction in Sox2^+^ cells within rhombomeres (Fig. 3Ce–h). Counting of Sox2^+^ cells in rhombomere 4 validated a greater than 6-fold decrease in Sox2^+^ cells upon treatment with L-mimosine, compared to the control (Fig. 3Dc). Moreover, the size of the rhombomeres seemed reduced upon L-mimosine treatment in comparison to controls (Fig. 3Cb,f; arrow). Quantification of the areas of r3 and r4 revealed an approximately 40 % reduction in L-mimosine-treated embryos compared to the control (Fig. 3Dd; n = 6 embryos in each group). These sets of experiments show that HB regions are enriched with slow-proliferative Sox2^+^ cells that can migrate and divide at their margins, allowing contribution of cells to rhombomeres.

### Neural differentiation at HBs

**Fig. 4:**
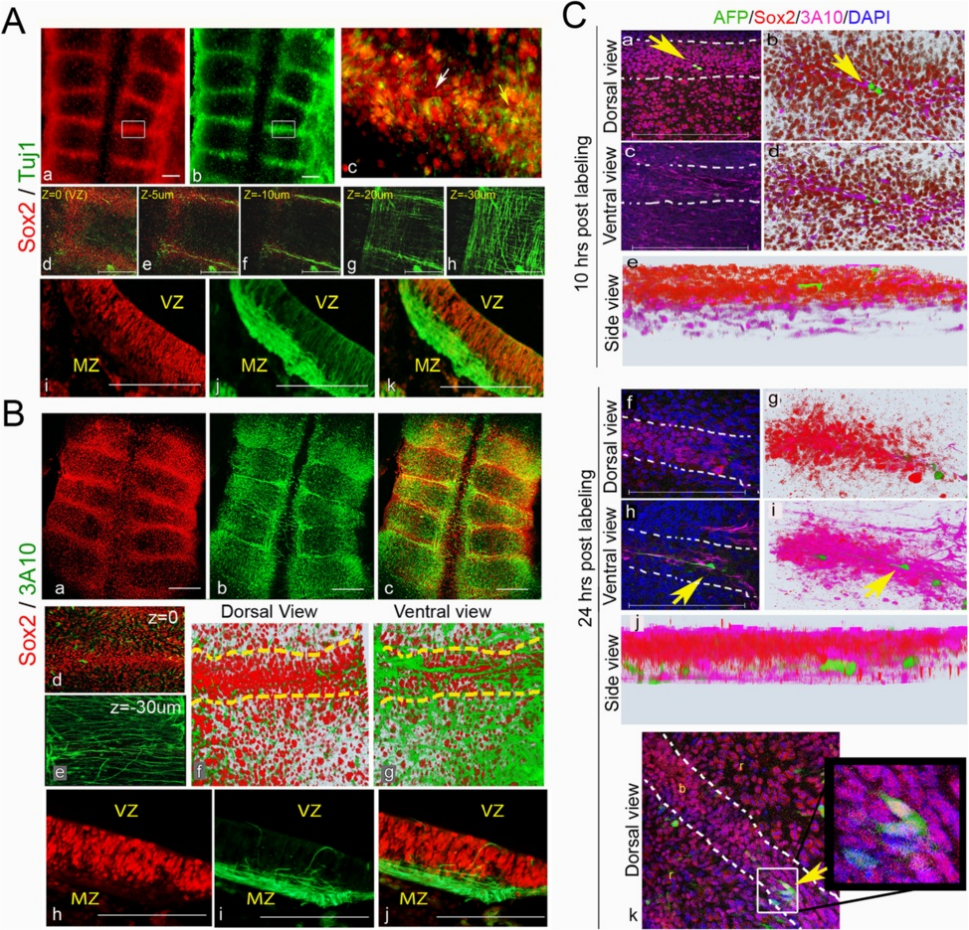
Neural differentiation at hindbrain boundaries. **A** Representative 18HH hindbrains immunostained for Sox2 and Tuj1 (n = 15). Flat-mounted views; high magnification of a boundary area marked in (**a**,**b**) is presented as a merged image in (**c**). Yellow and white arrows indicate a cell stained for both markers or for Sox2 alone, respectively. Sequential confocal Z-stack views from 0 to –30 μm of a boundary region (**d**–**h**). Transverse section of a boundary region shown in single (**i**,**j**) or merged (**k**) channels. **B** Representative 18HH hindbrains immunostained for Sox2 and 3A10 (n = 10). Flat-mounted views of single (**a**,**b**) or merged (**c**) channels. Confocal Z-stack merged channel images of a boundary at 0 (**d**) and –30 μm (**e**). 3D plots of boundary/rhombomere region from a merge channel images of dorsal (**f**) and ventral views (**g**) obtained from a confocal scan of 30 μm. Transverse section of a boundary region shown in single (**h**,**i**) or merged (**j**) channels. **C** Clonal analysis of AFP-injected boundary cells. Embryos (n = 10) were harvested 10 h (**a**–**e**) or 24 h (**f**–**k**) after treatment, stained for Sox2 and DAPI and analyzed as flat-mounts by confocal Z-stack images. Representative (**a**,**b**,**f**,**g**,**k**) dorsal views; (**c**–**d**,**h**–**i**) ventral views; (**e**,**j**) side views. (**b**,**d**,**e**,**g**,**i**,**j**) 3D models constructed from 30 Z-stacks shown in dorsal or ventral views, respectively. Yellow arrows indicate AFP^+^ cells. Dashed lines in B(f,g), C(a,c,f,h,k) indicates boundary–rhombomere intersection. VZ = ventricular zone, MZ = mantle zone, r = rhombomere, b = boundary. Scale bars = 100 μm

As a master gene in NSCs, Sox2 expression has to become downregulated upon differentiation. To reveal whether this pattern is recapitulated at HBs, Sox2 expression pattern was compared with Tuj1, a cytoskeletal protein expressed in differentiating neurons before or during terminal mitosis [93–96]. Flat-mounted views of st.18 hindbrains showed Tuj1 accumulation at HBs together with Sox2 (Fig. 4Aa–c, n = 15). Yet some, but not all, Sox2^+^ cells seemed co-labeled with Tuj1 (Fig. 4Ac, white arrow indicates a Sox2^+^/Tuj1^–^ cell and yellow arrow indicates a double-labeled cell). Confocal Z-stack analysis clarified this finding by showing that, while Sox2 is prominent at the alar layer and absent from the basal layer, Tuj1 is evident along the apical-basal domains (Fig. 4Ad–h), but its expression is most extensive in axonal fibers that stretch along boundaries (Fig. 4Af–g), in addition to its global axonal expression in rhombomeres (Fig. 4Ah). Similar results were seen in transverse sections, showing strong Sox2 expression at the ventricular/sub-ventricular layers but not the mantle zone at HBs, whereas Tuj1 is expressed in the processes of Sox2^+^ cells and enhanced at neural fibers that accumulate basally (Fig. 4Ai–k). Quantification of these stainings by flow cytometry revealed that out of the total (Sox2 + Tuj1) stained cells, approximately 60 % were Sox2^+^, approximately 13 % were Tuj1^+^ and approximately 25 % of stained cells were co-labeled with both (Additional file 7A). This indicates that around 40 % of the stained cells are differentiating whereas the rest of the stained tissue contains progenitorial Sox2^+^ cells. Staining for Sox2 and another neural differentiation marker, HuC/D, which labels RNA-binding proteins that are expressed in, and essential for, differentiating neurons [97], revealed a similar apical-to-basal pattern to that of Sox2 and Tuj1 in HBs (Additional file 7Bf,h,c). Similar to Tuj1 and HuC/D, the mRNA expression of other typical pan-neural differentiation markers, such as *NeuroD1*, *NSCL1* and *Brn3A* [98–100], were also found to be enhanced at HBs of st.18 embryos (Additional file 7Ba–e, g; n = 10 for each), further indicating active neurogenesis in these sites.

The marker 3A10 labels neurofilaments of fully differentiated neurons [100]. 3A10 was previously reported to accumulate at HBs [101]. Our whole-mount analysis confirmed the co-localization of Sox2 with 3A10 at the boundaries (Fig. 4Ba–c, n = 10). Yet confocal microscopy analysis (Fig. 4Bd,e) and transverse sections (Fig. 4Bh–j) of HB regions demonstrated the clear segregation of Sox2^+^ cells at the ventricular/sub-ventricular layer and 3A10^+^ fibers at the mantle zone, indicating an expected acquisition of this marker only upon completing neuronal migration. A 3D model of this staining was reconstructed from 50 Z-stacks between 0 and –30 μm, confirming the ventricular-to-mantle segregation of Sox2^+^ versus 3a10^+^ cells, respectively (Fig. 4Bf,g).

To reconcile the presence of both Sox2^+^ dividing cells and differentiated neurons at the boundaries, a clonal analysis was designed to monitor labeled boundary cells over time. A reporter (AFP) plasmid was electroporated into very few boundary cells, followed by harvest of the embryos after 10 h (to confirm AFP expression) or 24 h (to follow the migration of labeled cells; Additional file 7C shows the experimental scheme). At each time point, the hindbrains were stained for Sox2 and 3A10 and analyzed for the ventricular-to-mantle location of AFP^+^ cells (Fig. 4C, n = 10 embryos, >25 labeled cells/time point). Individual labeled cells were typically found at the apical surface of a Sox2^+^ boundary region after 10 h (Fig. 4Ca–e). In embryos harvested after 24 h, labeled cells migrated to the mantle area and adopted extended neuronal morphology with 3A10 expression (Fig. 4Cf–j). Some apical AFP^+^ boundary cells were also observed to remain in the VZ (Fig. 4Ck), further supporting the results shown in Fig. 3 regarding a dividing Sox2^+^ cell population at HBs. Collectively, these experiments support a typical differentiation pattern within HBs, with Sox2-expressing progenitors being located at the ventricular/sub-ventricular domains of HBs that give rise to early and late differentiating neurons in correlation with their migration and loss of Sox2 expression towards the mantle zone, while also retaining a presence of progenitors in the VZ.

### Formation of neurospheres with self-renewal and differentiation capacities from hindbrain-derived Sox2^+^ cells

**Fig. 5:**
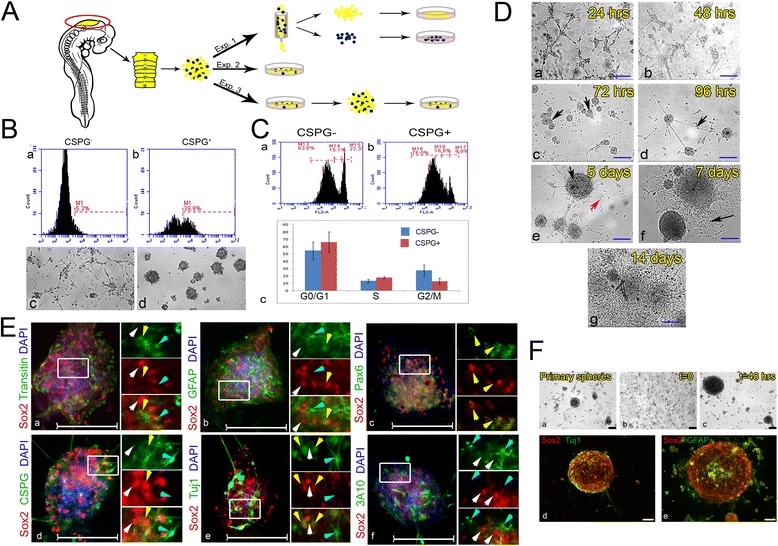
Formation of neurospheres with self-renewal and differentiation capacities from hindbrain-derived Sox2^+^ cells. **A** Scheme of experiments using primary cultures prepared from 18HH chick hindbrains. **B** Magnetic column-based separation of CSPG^+^ HB cells. Enrichment of CSPG^+^ compared to CSPG^–^ samples assayed by flow cytometry (**a**,**b**). Culture of CSPG^–^ and CSPG^+^ cells, respectively, shown in bright field 96 h post-separation (80 hindbrains used to obtain cell suspension) (**c**,**d**). **C** Cell cycle analysis by flow-cytometry on column-based separation of CSPG^+^ vs. CSPG^–^ cells that were stained with propidium iodide. CSPG^–^ and CSPG^+^ fractions, respectively (**a**,**b**). Graphic representation of cell cycle analysis results (**c**). Analysis was repeated twice, each time with three technical repeats (*P* value for each replica > 0.05, *t* test). **D** Bright-field views of primary cultures of hindbrain cells documented after plating from 24 h to 14 days (**a**–**g**). Arrows in (**c**) indicate newly formed spheres; in (**d**) an axon connecting two spheres; in (**e**) an intact sphere (black) with adjacent morphologically differentiating cell (red); and in (**f**) morphologically differentiated cells generated by collapsed sphere. **E** Co-staining of hindbrain-originated spheres (10 hindbrains used for each primary culture) for Sox2 with Transitin (**a**), GFAP (**b**), Pax6 (**c**), CSPG (**d**), Tuj1 (**e**), and 3A10 (**f**). High magnification views of the boxed areas are shown to the right of each panel in different channels. White, yellow or blue arrowheads indicate cells stained for Sox2 alone, for both markers or for the relevant marker alone, respectively. **F** Secondary sphere formation. Primary spheres (**a**) were dissociated and re-plated as single cells (**b**). Secondary spheres appeared after 48 h (**c**). Spheres were co-stained for Sox2 with Tuj1 (**d**) or GFAP (**e**). Scale bars in D,E = 100 μm, in F = 75 μm (43 hindbrains used to obtain primary spheres)

Cell culture systems are a common model to study NSCs [102, 103]. To determine whether the Sox2-expressing cells at HBs display typical NPC characteristics in vitro, HBCs from st.18 hindbrains were isolated (n = 80) from the rest of hindbrain cells and cultured in stem cell medium, which is commonly used to inhibit differentiation of presumed stem cell populations [104]. Based on the full co-localization of Sox2 with the membranous protein CSPG (Fig. 2D), we isolated live boundary cells using an anti-CSPG antibody on a magnetic immuno-column cell separation system [105] to segregate CSPG^+^ from CSPG^–^ hindbrain cells (Fig. 5A, exp.1). Flow cytometry analysis confirmed an approximately 8-fold increase (from 5.3 % to 39.9 %) in CSPG expressing cells in the eluted sample compared to the flow-through fraction (Fig. 5Ba,b). Culturing the cells for 7 days revealed a clear tendency of the CSPG^+^ fraction to form 3D spheres (Fig. 5Bd). These spheres were maintained in the culture for at least 2 months (data not shown). In contrast, the CSPG^–^ cells adhered to the plate and developed as a typical neuronal monolayer (Fig. 5Bc). Comparing the cell cycle profiles of these populations (Fig. 5C) showed an increased fraction of approximately 20 % of cells in G_0_/G_1_ and a decrease fraction of approximately 45 % cells in G_2_/M in the CSPG^+^ group, compared to the CSPG^–^ group (Fig. 5Ca–c; n = 50 embryos). These results provide a first in vitro support for the NPC-like features of the CSPG^+^ HBCs to form spheroid bodies in culture and to display a slower cell cycle, compared to inter-rhombomeric cells.

As the spheres or adhered neurons were obtained from separated cell fractions, we next tested whether culturing cells from the whole hindbrain together will give rise to both neurospheres and differentiating neurons. Primary cultures were prepared from freshly isolated st.18 hindbrains (Fig. 5A, exp. 2, n = 20), and grown in similar growth conditions as above. First, the growth of the cultured cells was monitored at subsequent days (Fig. 5D). During the first 3 days, the formation of discrete small aggregates was found (Fig. 5Da,b). These clusters of cells developed into 3D spheres that expanded and began to establish axonal connections with neighboring spheres over time (Fig. 5Dc–e; arrows), as expected from NSC spheroids in culture [102]. Single adherent cells with neuronal morphology also began to be evident on day 5 (Fig. 2De, red arrow). To support differentiation of the spheres, the medium in 5-day-old cultures was replaced with standard tissue culture medium. This treatment led to an extensive generation of a monolayer of cells with neuronal processes around the spheres (Fig. 5Df,g), consistent with the tendency of NSC spheres to differentiate into neurons in such conditions [106]. Notably, maintaining the culture in stem cell medium allowed retention of the spheres for up to 4 months (data not shown), further demonstrating the ‘stemness’ potential of the hindbrain-derived culture.

We next examined whether these spheroids express similar progenitorial and differentiated markers to those found in HBs in vivo (Figs. 2, 4). Strong expression of Sox2 was mostly evident in the cells within the spheres (Fig. 5Ea–f, n > 60 embryos), as expected from typical NPC-derived neurospheres. Co-staining of Sox2 with Transitin/GFAP/Pax6/CSPG revealed co-expression of these progenitorial markers in cells that constitute the spheres (Fig. 5Ea–d, n = 10 embryos for each marker). Noticeably, some cells stained solely for Transitin, GFAP or CSPG could also be found in the spheres. Comparing the expression of Sox2 with the neuronal differentiating marker Tuj1 revealed the existence of Sox2^+^ cells and Sox2/Tuj1-expressing cells, alongside cells with neuronal morphology which express Tuj1 only (Fig. 5Ee, n = 10 embryos). Staining with the neurofilament antibody 3A10 demonstrated the absence of cells co-expressing Sox2 and 3A10, while 3A10^+^ neurites were found extending from the sphere (Fig. 5Ef, n = 10 embryos). Overall, these hindbrain cultures recapitulate the specific expression patterns of HBCs that were observed in vivo (Figs. 2, 4), by demonstrating typical NPC characteristics of the Sox2^+^ spheres that can differentiate into neurons in vitro. These results are in agreement with data obtained from hindbrain-originated cells from human embryos, which have recently been shown to have NPC characteristics and a high neurogenic capacity in vitro, as well as the ability to retain their identity over a large number of passages [107].

Another key feature of NSCs is their capacity to self-renew. This ability is commonly determined in vitro by the regeneration of secondary spheres following dissociation of cells from the primary neurospheres [106]. To test this in our hindbrain-derived cultures, 4-day-old primary neurospheres were dissociated into single cells and re-cultured (Fig. 5A, exp.3, n = 43 embryos). The dissociated cells re-formed spheres within 3 days (Fig. 5Fa–c). The secondary spheres contained cells expressing Sox2, GFAP and Tuj1 (Fig. 5Fd,e), similar to the primary neurospheres. The self-renewal capacity of hindbrain-derived Sox2^+^ neurospheres further confirms the characteristic behavior of HB-derived cells as NPCs in vitro*.*

### Sox2 regulates neural differentiation within the developing hindbrain

**Fig. 6:**
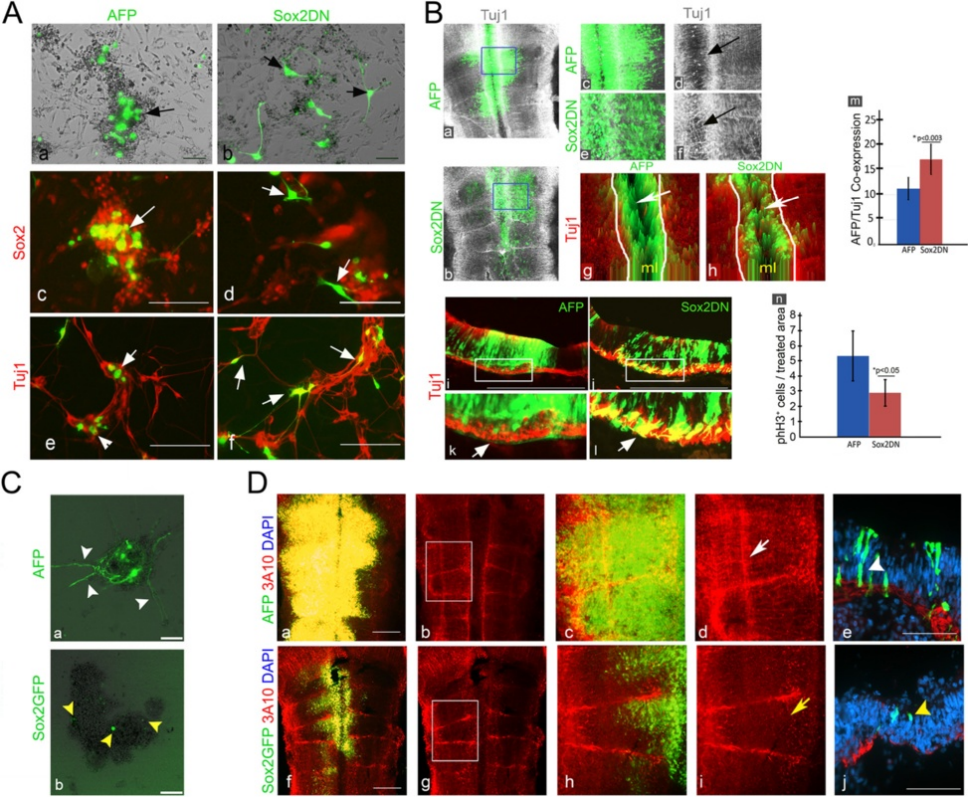
Sox2 regulates neural differentiation within the developing hindbrain. **A** Representative primary cultures prepared from 18HH hindbrains electroporated with control AFP (n = 12) or Sox2DN (n = 12) plasmids (green). Bright-field (**a**,**b**) and immunostaining with Sox2 (**c**,**d**) or Tuj1 (**e**,**f**). Arrows indicate electroporated cells. **B** Representative flat-mounts (**a**–**f**) (n = 10 in each treatment) and transverse sections (**i–l**) (n = 5 in each treatment) of 18HH hindbrains electroporated with AFP/Sox2DN (green) and stained for Tuj1 (grey/red). High magnification views of boxed areas in (**a**,**b**,**i**,**j**) are shown in **c**,**d**,**k** for AFP and in **e**,**f**,**l** for Sox2DN. Arrows in (**d**,**f**) indicate Tuj1-expression in the midline and in (**k**,**l**) indicate Tuj1^+^ fibers in the mantle zone. 2.5D plots obtained from flat-mounted hindbrains (**g**,**h**). Arrows indicate the midline. Flow cytometry quantification of hindbrains expressing AFP^+^ or Sox2DN^+^ cells that express Tuj1 (**m**). Quantification of phH3^+^ cells per area in AFP or SoxDN-treated hindbrains (**n**). **C** Primary cultures prepared from 18HH hindbrains electroporated with control AFP or Sox2GFP plasmids (green). Arrowheads indicate electroporated cells. **D** Representative flat-mounted views of AFP (n = 15) or Sox2GFP (n = 17) electroporated hindbrains stained for 3A10 (red) (**a**–**d**,**f**–**i**). Images in (**a**,**c**,**f**,**h**) show also AFP/GFP expressing cells. Enlargement of boxed regions in (**b**,**g**) is shown in (**c**,**d**,**h**,**i**). White and yellow arrows indicate typical and reduced 3A10^+^ fibers in rhombomeres, respectively. (**e**,**j**) Transverse sections of electroporated hindbrains. White arrowhead denotes AFP^+^ cell near the MZ; yellow arrowhead denotes Sox2GFP^+^ cell in the VZ. ml = midline. Scale bars = 100 μm

If Sox2^+^ cells in HBs act as reservoirs of NPCs, manipulation of Sox2 is expected to affect neural differentiation within the hindbrain. Loss-of-function experiments were performed using a dominant-negative form of Sox2 (Sox2DN), which contains the Sox2 High Mobility Group fused to the Engrailed repressor sequence. This construct was previously confirmed to act in a dominant negative fashion in the chick CNS [108]. Control (AFP) plasmid or Sox2DN:AFP (5:1 ratio) plasmids were electroporated into the hindbrains of st.15–16 embryos and harvested 24 h later. At these stages, the endogenous Sox2 expression tends to become downregulated from rhombomeres and maintained in HBs (Fig. 1A), such that upon the expression of the plasmids, Sox2 manipulation will primarily affect the boundary Sox2^+^ cells. Hindbrains were suspended to single cells and cultured in stem-cell medium for 48 h, to evaluate the contribution of the electroporated cells to the forming spheres (n = 12 embryos/treatment). Control AFP^+^ cells were mostly located within the spheres and displayed a rounded morphology, typical of cells that constitute neurospheres (Fig. 6Aa, arrow). In contrast, many of the Sox2DN-expressing cells grew long processes and were not located within the neurospheres, as expected from differentiating neurons (Fig. 6Ab, arrows). Immunostaining of the cultures for Sox2 or Tuj1 emphasized that many control cells expressed Sox2 rather than Tuj1 (Fig. 6Ac,e). Sox2DN^+^ cells showed less Sox2 expression and enhanced Tuj1 expression (Fig. 6Ad,f). Quantification of these data showed an increase of approximately 40 % in cells co-localized with Sox2DN^+^ and Tuj1, compared to control AFP^+^/Tuj1^+^ cells (Additional file 8Aa). Notably, some of the AFP^+^ cells also differentiated into neurons in the following 48 h, as expected in such cultures (data not shown). This in vitro experiment demonstrates a shift towards neural differentiation upon loss of Sox2 function.

To assess the effect of Sox2DN in vivo, embryos were electroporated and analyzed for Tuj1 expression. An organized pattern of Tuj1^+^ fibers was found in the hindbrain of AFP-treated embryos (Fig. 6Ba,c,d; n = 10, see also Fig. 4A). Embryos electroporated with Sox2DN showed distorted patterns of Tuj1-expressing axons compared to the controls, together with ectopic Tuj1 expression in the Sox2DN^+^ domains, such as in the floor plate (Fig. 6Bb,e,f; n = 10). Digital 2.5D images of the electroporated areas emphasized these results by demonstrating that AFP^+^ cells in the midline are largely excluded from areas of Tuj1 expression, whereas enhanced and disorganized Tuj1 expression is evident within the Sox2DN-expressing cells in the midline (Fig. 6Bg,h, arrows). Transverse sections (n = 5 embryos per treatment) supported these data by showing that Sox2DN-electroprated cells are found from the ventricular to the mantle zone, as expected at the time of fixation (Fig. 6Bj,l). Yet, enhanced localization of Sox2DN-expressing cells is clearly found within cells or axons in the mantle/Tuj1^+^ zone (Fig. 6Bj,l, arrow). This is in contrast to control AFP^+^ cells where fewer cells migrated this far basally and almost no AFP^+^ fibers could be detected (Fig. 6Bi,k). Quantifying these hindbrain cells by flow cytometry revealed a 1.6-fold increase in cells that co-expressed Sox2DN and Tuj1 (~18 %) compared to control AFP/Tuj1-expressing cells (~11 %; Fig. 6Bm). Analysis of HuC/D expression in electroporated embryos showed a similar effect of enhanced and aberrant distribution of HuC/D^+^ neurons upon Sox2DN expression compared to the typical organized pattern of this marker in control embryos (Additional file 8B).

As neurons differentiate, they exit the cell cycle. As electroporation of Sox2DN resulted in enhanced differentiation, embryos were tested for their cell proliferation state using phH3 staining. Embryos expressing Sox2DN showed fewer nuclei stained for phH3 within the electroporated cells compared to controls (Additional file 8 Da–d; n = 6 embryos each treatment). Quantification of these results confirmed a significant approximately 2-fold decrease in mitotic divisions of Sox2DN-expressing cells compared to AFP-expressing cells (Fig. 6Bn). This experiment further emphasizes the enhanced differentiation of Sox2DN^+^ cells in accordance with their lower cell proliferation.

The role of Sox2 was next tested by gain-of-function experiments, using a Sox2GFP expressing plasmid [109]. St.15–16 embryos were electroporated and confirmed to over-express Sox2 in the hindbrain, compared to control AFP (Additional file 8C). Notably, Sox2GFP electroporation did not yield ectopic Sox2 in all electroporated areas, as rhombomeres showed much less ectopic Sox2 expression compared to boundaries. This may imply that at st.18, boundary cells are more susceptible to Sox2 manipulation than rhombomeres. The effect of Sox2 misexpression was first examined in vitro by culturing hindbrain cells from AFP (n = 14) or Sox2GFP (n = 12) electroporated embryos for 8 days. These cultures formed neurospheres and grew neurons, as shown before (Fig. 5). Analysis of the electroporated cells revealed that AFP^+^ cells were found either within the neurosphere core as rounded cells or in its margins, where they acquired neuronal morphology (Fig. 6Ca, arrowheads). In contrast, most of the Sox2GFP^+^ cells were localized to neurospheres, where they remained small and rounded without sending out extensions (Fig. 6Cb, arrowheads). Quantification of the percentage of GFP^+^ neurites in relation to GFP^+^ cells in each group showed a significant reduction in the Sox2GFP group compared to the control (Additional file 8Ab).

Examination of the differentiation state of the cells was next performed in vivo by staining embryos for 3A10. Whole-mount views revealed a characteristic pattern of 3A10 expression in hindbrain axons of AFP-treated embryos (Fig. 6Da–d; n = 15), similar to untreated embryos (Fig. 4b). In contrast, Sox2GFP-treated embryos showed an overall marked reduction in 3A10^+^ neurons, including but not only in electroporated areas (Fig. 6Df–i; n = 17). Transverse sections confirmed these findings and emphasized that Sox2GFP^+^ cells tend to remain in the ventricular zone, while AFP^+^ cells migrate throughout the ventricular-to-mantle axis (Fig. 6De,j). Assessment of phH3^+^ nuclei in the electroporated cells revealed an approximately 50 % increase in the presence of dividing cells that co-express Sox2GFP as compared to AFP (Additional file 8De; n = 11 for each treatment).

Overall, the data provided here suggest that inhibition of Sox2 enhances the differentiation and reduces the proliferation state of the cells, while ectopic expression of Sox2 leads to the sustainment of non-differentiating cells at the ventricular layer where hindbrain cell proliferation occurs. These findings demonstrate the importance of boundary-enriched Sox2^+^ cells in the direct regulation of neural differentiation in the chick hindbrain.

## Discussion

**Fig. 7:**
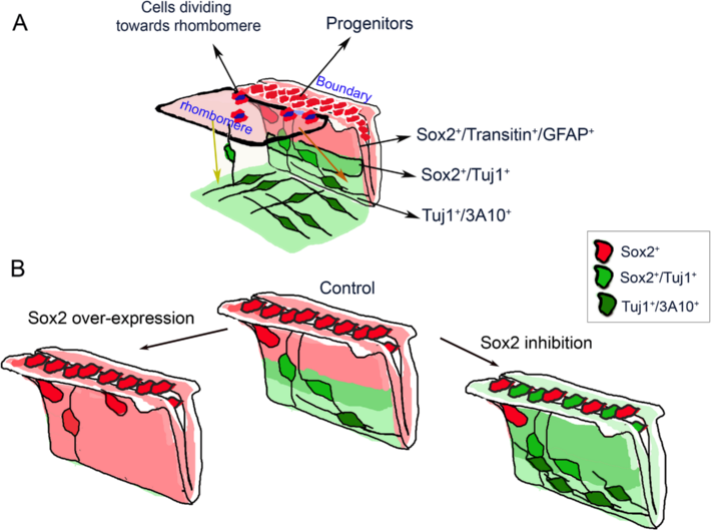
A model of the distribution of boundary cells in the normal st.18 hindbrain and upon Sox2-manipulation. **A** A scheme emphasizing the distribution of Sox2^+^ cells at st.18 hindbrain. According to our model, boundaries consist of slow dividing Sox2^+^/Transitin^+^/GFAP^+^ progenitors. At the boundary–rhombomere intersection Sox2^+^ cells divide towards the adjacent rhombomeres. Hindbrain neural differentiation occurs on the ventricular-to-mantle axis as cells lose progenitor markers and acquire neural markers (Tuj1/3A10) during migration to mantle zone. **B** Schematic summary of the impact of Sox2 manipulation on hindbrain neural differentiation: Sox2 overexpression leads to an increase in Sox2^+^ cells at the ventricular layer and reduced neural differentiation, whereas inhibition of Sox2 leads to enhanced and aberrant neural differentiation

Seminal studies have previously identified the segmentation of the hindbrain into lineage-restricted rhombomeres and the formation of inter-rhombomeric boundaries as unique cellular domains that are evolutionarily conserved [7, 40, 64, 110]. Yet the function of HBs remained unclear. The data found in this study reveals novel aspects regarding the molecular properties of HBs, their spatial and temporal organization, and their functions as repetitive pools of NPCs, which play a central role in the neuronal organization of the hindbrain in a Sox2-dependant manner (Fig. 7).

The expression of Sox2 was found to be highly dynamic – initially, Sox2^+^ cells appear uniform in the whole hindbrain, but as development proceeds, they become restricted to HBs. We and others have found (previously or in the current study) a similar trend in other progenitorial/stem cell markers in the chick or mouse hindbrain (i.e., Pax6, Transitin, FGF3, follistatin, PLZF1, Id1) [15, 39, 41, 65, 66, 72, 111, 112], suggesting a spatio-temporal shift in the control of neural differentiation in the hindbrain from broad domains to more restricted zones. The fact that each rhombomere exhibits a unique genetic profile and neural fate, whereas all boundaries share similar non-neurogenic properties at st.18, further emphasizes the molecular/fate distinction between these domains, and supports the scenario of the boundaries as repetitive pools of stem-like cells at stages when rhombomeres are actively differentiating. The tendency of progenitorial domains to converge to HBs resembles the situation in the nearby MHB. The expression of the bHLH transcription factors Her5 (in zebrafish) or Hes1/3 (in mice), two genes that act to inhibit neural differentiation of progenitor cells, shrinks from the entire MH of the early embryo to the MHB at older stages [28, 29, 33]. The role of Her/Hes to maintain the MHB as a non-neurogenic zone was found to be mediated via the repression of pro-neural genes, and by this to contribute to the integrity of the MHB and the balance between growth and differentiation in the midbrain and anterior hindbrain [30, 113–115]. The Her5^+^ cells in the MHB were also found to be maintained as reservoirs of NSCs in the adult zebrafish. Intriguingly, a previous study has reported that *Hes1* is expressed in the boundaries of the mouse hindbrain, where it was involved in the segregation of HBCs from rhombomeric cells [42]. In a parallel manner, the Notch pathway-related gene *Radical Fringe* was described to be expressed in zebrafish HBs and needed for boundary formation [40]. Future studies will be required to uncover whether *Hes* genes, or other Notch-related factors, are active in HBs downstream or upstream of Sox2, to establish and maintain these domains as non-neurogenic zones.

Which mechanisms might regulate the rhombomeric downregulation of Sox2 and its maintenance in HBs? We have previously found a similar rhombomere-to-boundary shift in *FGF3* and *follistatin*, which was controlled by a secreted signal from boundary cells to downregulate these genes in rhombomeres [43]. Although the identity of this factor(s) is not known, redeployment of the same boundary-originated molecular machinery to also downregulate rhombomeric Sox2 is possible as part of a general mechanism to narrow down domains of NSCs during embryonic maturation. Furthermore, multiple studies in different types of stem cells or cancer cells have shown that repression of Sox2 transcription can be mediated directly by cell cycle effector genes or micro-RNAs [116–119], or by epigenetic modulations (i.e., phosphorylation, methylation, ubiquitination) that lead to Sox2 downregulation [120–124]. Whether such mechanisms may be differentially active in rhombomeres or boundaries remains unknown.

Other possible mechanisms to restrict the Sox2^+^ domains to the boundaries may arise from cell-surface differences between boundaries and rhombomeres – as the ECM molecule CSPG is exclusively expressed at HBs, it may mediate the consistency of these domains. Indeed, this proteoglycan is present in several other NSC microenvironments where it was suggested for NSC survival and the maintenance of their niches [84, 125–127]. The rhombomere-exclusive expression of the cell surface glycoconjugate Hnk-1 may also contribute to the establishment of Sox2 negative/positive zones in the hindbrain, as in the spinal cord inhibition of Sox2 that led to ectopic expression of Hnk-1 [56]. Hence, it is possible that the opposite distribution of Sox2/CSPG in HBs and Hnk-1 in rhombomeres is necessary to stabilize the HB territories.

Within the boundary, two subgroups of Sox2^+^ cells were found, one slower and one faster dividing, which are positioned in the middle or edges of the boundaries, respectively. We also found different migration/differentiation properties of Sox2^+^ cells in the boundaries – some display a loss in Sox2 along the ventricular-to-mantle axis and acquisition of neuronal fates, while others remain in the ventricular layer, and another group migrates laterally to provide Sox2^+^ dividing cells to the rhombomeres. Distinct populations of Sox2^+^ NSCs were found in other sites, such as in the subventricular zone of the hippocampus. One quiescent subgroup displays radial glia-like morphology with a long process across the granular layer and serves as reservoir of uncommitted NSCs, while the second proliferating group lack radial processes and are multipotent and self-renewing NPCs [86, 88, 128]. On the other hand, spinal cord Sox2^+^ cells are more homogenous in their active proliferation state, directed apical-to-basal differentiation and neuronal versus glial fates in earlier stages of development [61, 129, 130]. Exploring the mechanisms that control the occurrence of the different Sox2^+^ subgroups and the fate of each subpopulation at later stages of development awaits further research.

## Conclusions

In this study, we provide new insights to an old open question regarding the role of hindbrain boundaries. Evidently from this work, hindbrain boundaries consist of Sox2+ cells at their ventricular zone that hold two main roles; the first is to provide Sox2+ proliferating progenitor cells to adjacent rhombomeres while the second is to give rise differentiating neurons into the mantle layer of boundaries in a Sox2-dependent regulation. As compartment boundaries are found in different tissues throughout evolution, and since hindbrain development is well-conserved in all vertebrates, our data from the chick embryo model helps to better understanding the biology of boundaries and their contribution to CNS development.

## Methods

### Embryos

Fertile Loman Broiler chicken eggs (Gil-Guy Farm, Moshav Orot, Israel) were incubated at 38 °C until embryos reached the required Hamburger Hamilton stage. Following manipulations, embryos were fixed in 4 % paraformaldehyde (PFA; Sigma-Aldrich, USA) and stored at 4 °C.

### In ovo electroporation

Plasmids used were pMES-Sox2-IRES-GFP (Sox2-GFP) [109], pCMV/SV1-cSox2HMG-Engrailed (Sox2DN) [108] and pCIG-IRES-GFP (AFP). A list of plasmids is presented in Additional file 9. Plasmids were diluted in TE buffer to working concentrations between 1.5 and 4 μg/μL. Plasmids were injected into the hindbrain lumen of st.14–15 embryos using a pulled glass capillary. Electroporation was performed using L-bent gold electrodes (1 mm diameter) in a parallel holder and an ECM 830 electroporator (BTX, Harvard Apparatus, USA) using four 45-millisecond pulses of 18–20 volts with pulse intervals of 300 milliseconds [34]. Embryos were incubated up to st.18 prior to harvesting.

### Primary cell cultures

Hindbrain primary cell cultures were prepared from hindbrains dissected from st.18 embryos. Hindbrains were incubated for 10 minutes at 37 °C with TrypLE Express (Gibco, USA) to dissociate the tissue into single cells, then TrypLE was neutralized with 10:1 standard cell culture medium containing DMEM/F-12 1:1 with 10 % fetal bovine serum, Penicillin-Streptomycin (Pen-Strep, 1:50; Gibco, USA) and Amphotericin B (1:400; Sigma-Aldrich, USA). Cells were passed through a 100-μm mesh strainer and centrifuged at 600 *g* for 10 minutes. Excess medium was carefully removed and cells were plated in a 24-well Nunclon Delta Surface culture plate (Thermo Scientific, USA) with standard medium or stem cell medium containing DMEM/F-12 1:1 with 20 % KnockOut serum replacement, GlutaMax L-alanyl-L-glutamine (2 mM), non-essential amino acids (0.1 mM; all from Gibco, USA), β-mercaptoethanol (0.1 mM; Sigma-Aldrich, USA), PenStrep (1:50) and Amphotericin B (1:400). Cell cultures were incubated at 37 °C/5 % CO_2_ and culture media were replaced every 48 h. For replating experiments, cells were dissociated with TrypLE and centrifuged as described above, then plated with both types of media.

### Magnetic bead cell separation

Cell separation experiments were carried out using the MACS micro-beads cell separation system (Miltenyi Biotec, Germany) according to the manufacturer’s protocol. Briefly, isolated live hindbrain cells from st.18 embryos were incubated with primary anti-CSPG antibody for 30–60 min at room temperature. After washing, cells were incubated with anti-Mouse IgG micro-bead-conjugated secondary antibody solution (Miltenyi Biotec, Germany) for 30 min at 4 °C. Cells were then washed and passed through MACS cell separation magnetic columns placed on MACS iMAG separator (Miltenyi Biotec, Germany). Cells were eluted from the column by removal from the magnetic field and application of mild force. Separated cell populations were plated in DMEM/F12 cell culture medium and incubated at 37 °C/5 % CO_2_. Successful separation was analyzed by the addition of anti-Mouse Alexa-Fluor 488 antibody for the last 5 min of secondary antibody incubation. Quality control analysis was performed using BD Accuri C6 flow cytometer. Data analysis and gating were performed using the BD Accuri C6 software.

### Immunofluorescence

For whole-mount staining, fixed embryos were washed and incubated in PBS with 0.1 % Tween20/5 % goat serum (Biological Industries, Israel) for 2 hours at room temperature for blocking, before incubation overnight with the following antibodies: Mouse anti-3A10 (3A10, DSHB, University of Iowa, USA), Mouse anti-Transitin (EAP3, DSHB, University of Iowa,USA), Mouse anti-Pax6 (PAX6, DSHB, University of Iowa, USA), all hybridoma bank antibodies used in concentration of 1:50. Mouse-anti CSPG (1:50; # c8035; Sigma-Aldrich, USA), Mouse-anti CD57 (1:400; #560844; BD Biosciences, USA), Rabbit-anti Sox2 (1:400; #ab5603; Millipore, USA), Mouse anti-Tuj1 (1:400, #ab14545; Abcam, UK), Mouse anti-GFAP (1:400; # IF03L; Calbiochem, USA), Rabbit anti-phH3 (1:400; #sc-8656-R; Santa Cruz Biotechnology, USA) or Mouse anti-HuC/D (1:400; #A21271; Molecular Probes, USA). Following washes, embryos were incubated for 2 h at room temperature or overnight at 4 °C in PBS/0.1 % Tween20/5 % goat serum with secondary antibodies: Goat anti-mouse Alexa488, Goat anti-mouse Alexa594, Goat anti-rabbit Alexa488, or Goat anti-rabbit Alexa594 (all 1:400; Molecular Probes, USA), washed again, and incubated for 10 minutes at room temperature in PBS with DAPI (1:1000; Sigma-Aldrich, USA). Hindbrains were mounted on slides as flat-mounts (ventricular side facing up). For frozen section staining, fixed embryos were incubated overnight in PBS/30 % sucrose (Sigma-Aldrich, USA) at 4 °C. Embryos were then embedded and frozen in Optimal Cutting Temperature compound (Sakura Finetek, USA) in a cryomold. Blocks were sectioned at 10-μm thickness using a CM1860 cryostat (Leica, Germany) and mounted onto slides. Slides were washed, blocked and stained as described above for whole embryos. For cell culture staining, culture medium was carefully removed from the wells and cells were fixed in 4 % PFA solution for 15 minutes at room temperature. Cells were washed for 5 minutes with PBS/0.01 % Triton X-100 (Sigma-Aldrich, USA) and again for 5 minutes with PBS twice. Cells were incubated for 2 h at room temperature or overnight at 4 °C in PBS/1 % BSA (Sigma-Aldrich, USA) with primary antibodies as described above. Following PBS washes, cells were incubated for 2 h at room temperature or overnight at 4 °C in PBS/1 % BSA with appropriate Alexa-Fluor secondary antibodies (1:500). Cells were washed and incubated with DAPI (1:1000) for 10 minutes, washed again and kept in PBS at 4 °C until optical analysis. In some experiments, a round glass cover slip was placed inside each well to flatten the spheres for better imaging.

### In situ hybridization

Whole-mount in situ hybridization was performed as described previously [131], using probes for chick *NSCL1* (chick EST clone 474 F24, BBSRC, UK), *Brn3a* (a gift from A. Graham) and *NeuroD* (a gift from D.Schultea). The DIG labelled probes were detected using NBT/BCIP as substrate (Roche, Basel Switzerland), as described previously [72, 34]. List of plasmids is presented in Additional file 9.

### Flow cytometry

Hindbrains were harvested and dissociated into single cell suspensions as described above. Following enzymatic dissociation, cells were fixed in 4 % PFA for 30 min at room temperature and centrifuged at 600 *g* for 10 minutes. Cells were washed in PBS/0.01 % Triton X-100 for 10 minutes and re-centrifuged, followed by incubation overnight at 4 °C in PBS/1 % BSA with appropriate primary antibodies (1:300). Following washes and centrifugation, cells were incubated for 2 h at room temperature in PBS/1 % BSA with the Alexa-Fluor secondary antibodies (1:300), washed and re-centrifuged as described above. For cell cycle analysis, cells were fixed in 70 % ethanol overnight at 4 °C, washed and stained with propidium iodide (10 μg/mL; Sigma-Aldrich, USA) for 20 minutes on ice. Cells were next suspended in PBS and passed through an Accuri C6 Flow Cytometer (BD Biosciences, USA). Data analysis and gating were performed using the BD Accuri C6 software.

### Clonal analysis and CM-DiI labeling experiments

For clonal analysis, embryos of st.15 had their roof plate carefully opened. Transfection mix (10 % glucose in 1XPBS, 0.1 μL Turbofect in vivo (Thermo Scientific, USA) containing 1.5 μg AFP plasmid was directly injected to a few hindbrain boundary cells using a fine-pulled glass capillary. Transfection embryos were then electroporated as described above. Next, embryos were incubated for 10–24 h prior to harvesting. Harvested embryos were fixed and stained with Sox2 and 3A10 antibodies and their hindbrains were flat-mounted as described above. For CM-DiI labeling, CM-DiI (C-7000, Molecular Probes) was dissolved in 100 % ethanol to a concentration of 1 mg/mL, which was then further diluted 1:10 in DMSO to working concentration of 10 μg/mL. Labeling was performed in ovo by directly injecting CM-DiI into the hindbrain of st.15 embryos. Hindbrains were harvested 24 h later, fixed and placed on slides for microscopic observation.

### Time lapse analysis

St.18 embryonic hindbrains were isolated and cleaned from external tissues. After opening of the roof plate, hindbrains were incubated for 10–20 min in hESC medium supplemented with 1 μL of 0.5 mg/mL Hoechst bisbenzimide 33258 (Sigma-Aldrich, USA), after which they were placed on cover slips embedded with rat tail collagen (2 mg/mL, Sigma-Aldrich, USA) and covered with 200 μL hESC medium. In some experiments, a few boundary cells were also labeled by injection of CM-DiI (C-7000, Molecular Probes, USA). CM-DiI was dissolved in 100 % ethanol to a concentration of 1 mg/mL, and diluted 1:10 in DMSO to working concentration of 10 μg/mL. Hindbrains were incubated in a closed chamber at 38 °C/5 % CO_2_ for approximately 2 h and in vivo images were taken every 30 seconds using a Leica inverted confocal SP1 microscope (Leica, Germany) with W-Plan Apochromat × 10 objective. In some experiments, similarly treated hindbrains were fixed with soft agar and saturated with hESC medium, after which in vivo imaging was performed every 250 seconds for 6–8 h using a Zeiss LSM 780 upright confocal microscope (Carl Zeiss, Jena, Germany) with a W-Plan Apochromat × 20 objective.

### L-mimosine assay

L-mimosine treatment was performed by placing AGX‐100 beads soaked with either 0.5 mM L-mimosine (Sigma-Aldrich, USA) or DMEM/F12 as control into the hindbrain lumen of st.15 chick embryos. Embryos were incubated for 6 h, followed by bead removal and harvesting of embryos. Embryos were immunostained as described above.

### Time course Cm-DiI injection

CM-DiI (C-7000, Molecular Probes) was dissolved in 100 % ethanol to a concentration of 1 mg/mL, which was then further diluted 1:10 in DMSO to working concentration of 10 μg/mL. Labeling was performed in ovo by directly injecting CM-DiI into the hindbrain of 13HH chick embryos. Hindbrains were harvested 24 h later, fixed and placed on slides for microscopic observation.

### Data analysis and imaging

Cell cultures and whole-mounted tissues were imaged using CTR 4000 confocal microscope with DFC300FXR2 camera (Leica, Germany). Z-stack images were generated using Leica Microsystems software. Slides were imaged on Eclipse E400 microscope (Nikon, USA) using DP70 camera (Olympus, Japan), or on Axio Imager M1 microscope using AxioCam MRm camera (both from Zeiss, Germany). In some cases, Leica LAS-AF image analysis software was used to generate 2.5D plots or 3D plots. Time-lapse movie files were composed of 4/6 frames/sec using Leica LasX software and processed in Adobe premiere software.

Quantification of the relative distribution of Sox2^+^ cells in boundaries versus rhombomeres was determined by calculating the ratio of Sox2^+^ cells in areas of 100 DAPI^+^ nuclei using ImageJ software (NIH, USA.) Sox2^+^ cells were counted in eight regions randomly selected in r4/5 and the adjacent boundaries. The number of Sox2^+^ cells was divided by number of DAPI^+^ nuclei in order to obtain the Sox2/DAPI ratio for each examined region. To quantify the relative distribution of phH3^+^ cells in boundaries versus rhombomeres, the number of phH3^+^ cells was counted using ImageJ in 20 comparable areas of 100 × 20 μm. Randomly selected areas in r2-4/b3-5 in seven embryos were used for the purpose of this analysis. Quantification of Sox2^+^ mitotic cells was performed by marking three comparable regions of boundary, boundary/rhombomere interface and rhombomere from Sox2/DAPI-stained hindbrains, using ImageJ. The number of Sox2^+^ dividing cells was divided by the size of the selected field to acquire the occurrence rate of Sox2^+^ mitotic cells in each examined area. Quantification of Sox2^+^ cells in the L-mimosine assay was performed by counting the number of Sox2^+^ cells in r4 of treated versus control embryos in ImageJ. Quantification of rhombomere sizes was performed using measurement tools of ImageJ 1.410, and normalized to control.

Quantification of cells in culture was performed by counting electroporated cells that were co-labeled with antibodies or formed neurites, using ImageJ. Quantification of phH3^+^ cell in Sox2-manipulated cells was performed by selecting electroporated regions in 4 to 5 embryos from each group using ImageJ software. The number of phH3^+^ cells was divided by the areas of the selected field to acquire the occurrence rate of phH3 in selected areas. In all cases, statistical analysis and T-tests were performed using Microsoft Excel software (version 2013).

## Additional files

Additional file 1: Confocal analysis of st.18 hindbrains. Confocal analysis if 18HH hindbrain stained with DAPI. (a–d) Sequential Z-stack from 0 to –12 μm of a boundary/rhombomere area. (e–f) Confocal-generated 3D model; higher magnification of boxed area in (e) is shown in (f). Hindbrain boundaries are denoted by dashed lines. r = rhombomere, b = boundary; VZ = ventricular zone; SVZ = sub-ventricular zone. (TIF 1891 kb) Additional file 2: Expression of progenitor markers in the hindbrain. A. Typical flow cytometry plots for 18HH hindbrain cells stained with the progenitor markers Transitin and CSPG antibodies. (b,e) Control samples stained with secondary Ab only. (c,f) Samples stained for Transitin and CSPG, respectively. B. Representative flat-mounted views of hindbrains of 15HH chick embryos stained of progenitor markers. Hindbrains were immunostained for Sox2 with Pax6 (a-c) and Transitin (d-f) (n = 10/marker). Red/green or merged channels are shown in images (a-f), respectively. Scale bars = 100 μm. (TIF 1062 kb) Additional file 3: Time lapse analysis of Hoechst-stained chick hindbrain. First segment presents low magnification view of the documented hindbrain and states the magnified region of the second segment of the movie. Second part presents contribution of cells from the boundary to the adjacent rhombomere by cell division at the boundary–rhombomere intersection (white/orange arrows) and by cell migration from the boundary region towards the rhombomere (pink arrow). (MP4 10833 kb) Additional file 4: Time lapse analysis of Hoechst-stained hindbrain that was labelled with CM-DiI in the boundary region. Labeled cells are shown to move from the boundary towards the rhombomere (arrow). (MP4 16163 kb) Additional file 5: Time lapse analysis of CM-DiI-labeled cells in a boundary region of chick hindbrain explants. Labeled cells are shown to migrate from the boundary towards the rhombomere (left, arrow) or to divide towards the adjacent rhombomere (right, arrow). Some cells remain still within the boundary during the entire documentation. Tissue was stained with Hoechst prior to microscopy analysis. (WMV 4457 kb) Additional file 6: CM-DiI labeling of boundary and rhombomere cells. Representative flat-mount confocal views of CM-DiI labelled rhombomere (a,b) or boundary (c,d) (n = 5 hindbrains). Arrows indicate injection site, yellow lines indicate boundaries. Outlined areas in (b,d) show dye expansion. (TIF 11336 kb) Additional file 7: Expression of neural-differentiation markers in the hindbrain. A. (a) Representative flow cytometry plot from 18 HH hindbrain stained with Sox2 and Tuj1. Quantification of relative abundance of Sox2/Tuj1-expressing cells is shown. (b) Graphic representation of Sox2/Tuj1 distribution as percentage of total stained cells. B. Representative flat-mounted views of 15HH and 18HH hindbrains in situ hybridized with RNA probes against *NeuroD*, *NSCL1* and *Brn3a*, or immunostained for HuC/D (n = 10/marker) (e-h). Expression of *NSCL1* and HuC/D shifts from punctuated rhombomeric expression in 15HH to boundary-enhanced expression at 18HH. C. (a-c) Representative flat-mounted views of 18HH hindbrains stained for Sox2 and HuC/D (n = 10). Merged image is shown in (c). (d-g) Sequential Z-stack analysis from 0 to –20 μm of a boundary area. Arrows indicate site of neural differentiation. Scale bars = 100 μm. D. Scheme of the clonal-analysis of HB cell-labeling experiment using injection of AFP plasmid into single cells and harvesting at two time points. (TIF 4496 kb) Additional file 8: Effects of Sox2 manipulation on hindbrain neural differentiation and cell proliferation. A. (a) Quantification of co-localization of Tuj1 with Sox2DN or AFP-expressing cells in primary hindbrain cultures (n = 11 hb/treatment). (b) Quantification of neurites expressing AFP (n = 14) or Sox2GFP (n = 12) plasmids in primary hindbrain cultures. B. (a-f) Representative flat-mounts views of 18HH hindbrains electroporated with AFP/Sox2DN (green) and stained for HuC/D (grey) (n = 5/treatment). High magnification views of boxed areas in (a,b) are shown in (c,e) for AFP and in (d,f) for Sox2DN. Yellow arrows indicate aberrant HuC/D expression. (g,h) 2.5D plots obtained from confocal analysis of flat-mounted hindbrains. White arrows indicate the midline. C. Representative flat-mounts of 18HH hindbrains electroporated with AFP (a,b) or Sox2GFP plasmids (green) and stained for Sox2 (red; n = 10). White arrows indicate areas of high Sox2 expression in electroporated HBs, yellow arrow indicates weaker upregulation of Sox2 in electroporated rhombomere. D. (a–d) Representative flat-mounts of 18HH hindbrains electroporated with AFP/Sox2DN plasmids (green) and stained for phH3 (red) (n = 5/treatment). Higher magnifications of boxed areas in (a,b) is shown in (c,d). Arrows in (a,b) indicate boundary regions. Arrows in (c,d) indicate electroporated cells with or without phH3, respectively. (e) Quantification by cell-counting of phH3^+^ cells per area in hindbrains electroporated with AFP or Sox2GFP plasmids (n = 11 hb/treatment). ml = midline. Scale bars = 100 μm. (TIF 2775 kb) Additional file 9: Detailed description of clones and plasmids used in this study. (DOCX 13 kb)
