# Supplementary figures and images for: A new role of hindbrain boundaries as pools of neural stem/progenitor cells regulated by Sox2
Source: BMC Biol. 2016 Jul 8;14:57. doi: 10.1186/s12915-016-0277-y (PMC4938926; doi:10.1186/s12915-016-0277-y)

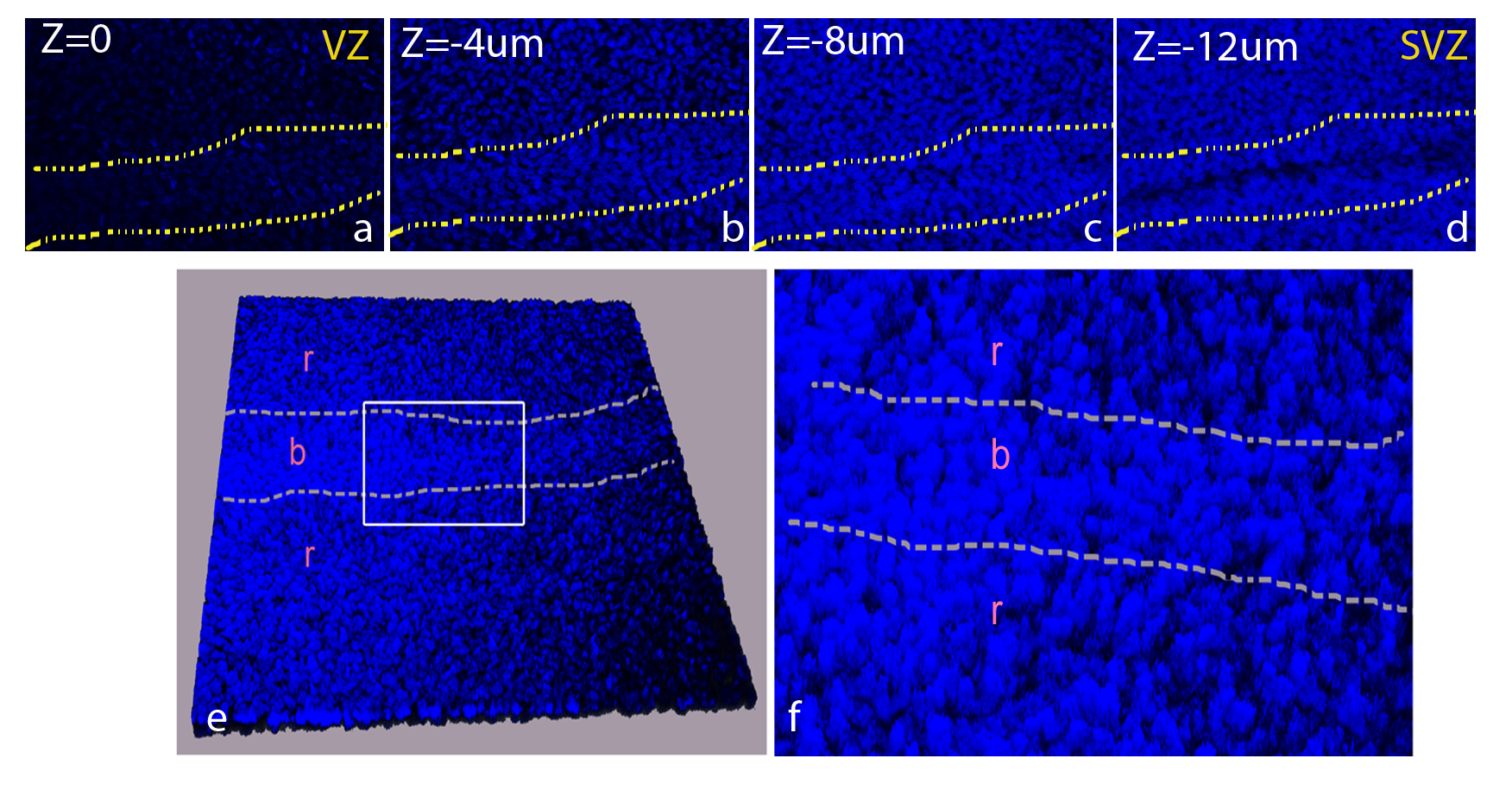

Supplement: Additional file 1: — Confocal analysis of st.18 hindbrains. Confocal analysis if 18HH hindbrain stained with DAPI. (a–d) Sequential Z-stack from 0 to –12 μm of a boundary/rhombomere area. (e–f) Confocal-generated 3D model; higher magnification of boxed area in (e) is shown in (f). Hindbrain boundaries are denoted by dashed lines. r = rhombomere, b = boundary; VZ = ventricular zone; SVZ = sub-ventricular zone. (TIF 1891 kb) [file 12915_2016_277_MOESM1_ESM.tif]

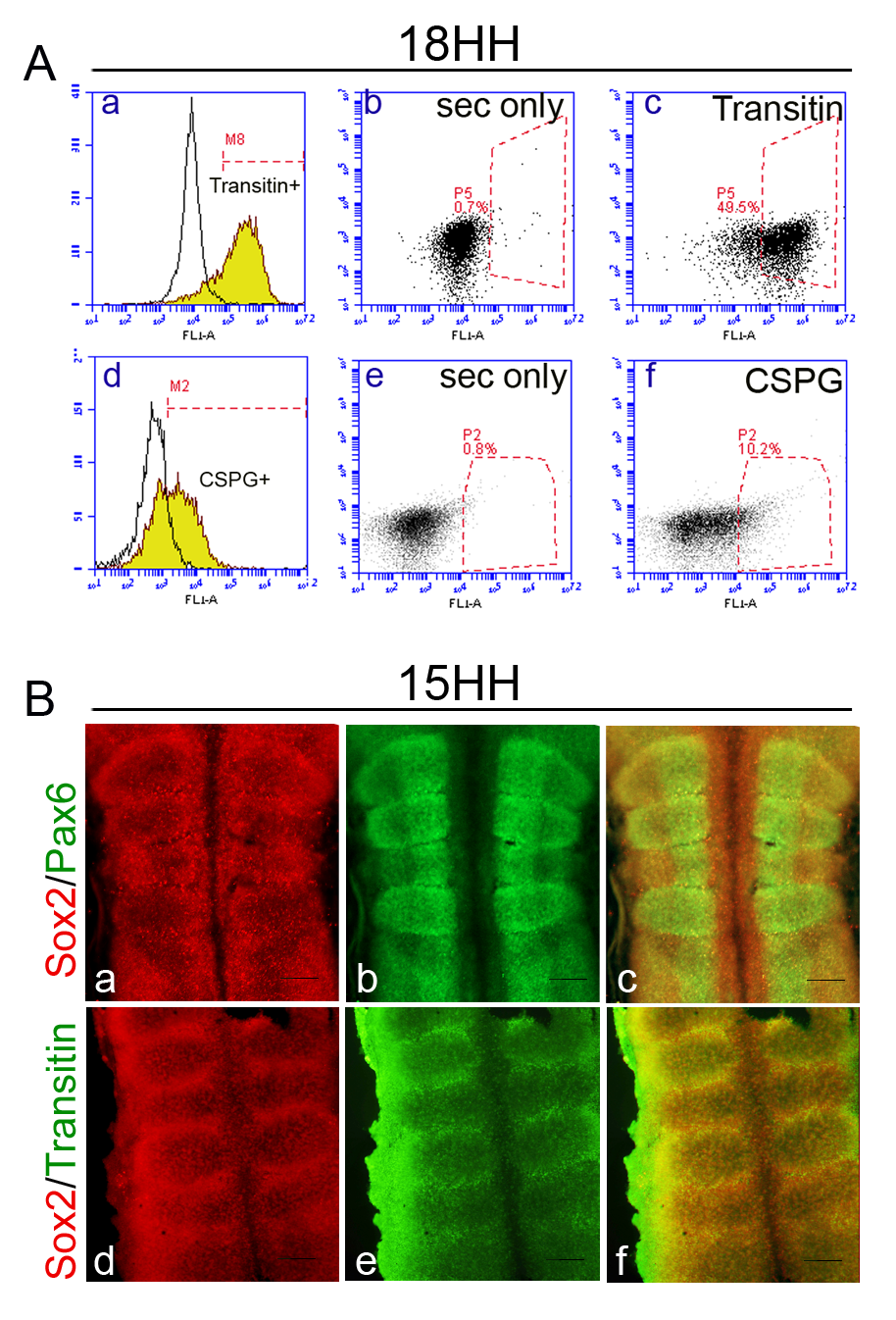

Supplement: Additional file 2: — Expression of progenitor markers in the hindbrain. A. Typical flow cytometry plots for 18HH hindbrain cells stained with the progenitor markers Transitin and CSPG antibodies. (b,e) Control samples stained with secondary Ab only. (c,f) Samples stained for Transitin and CSPG, respectively. B. Representative flat-mounted views of hindbrains of 15HH chick embryos stained of progenitor markers. Hindbrains were immunostained for Sox2 with Pax6 (a-c) and Transitin (d-f) (n = 10/marker). Red/green or merged channels are shown in images (a-f), respectively. Scale bars = 100 μm. (TIF 1062 kb) [file 12915_2016_277_MOESM2_ESM.tif]

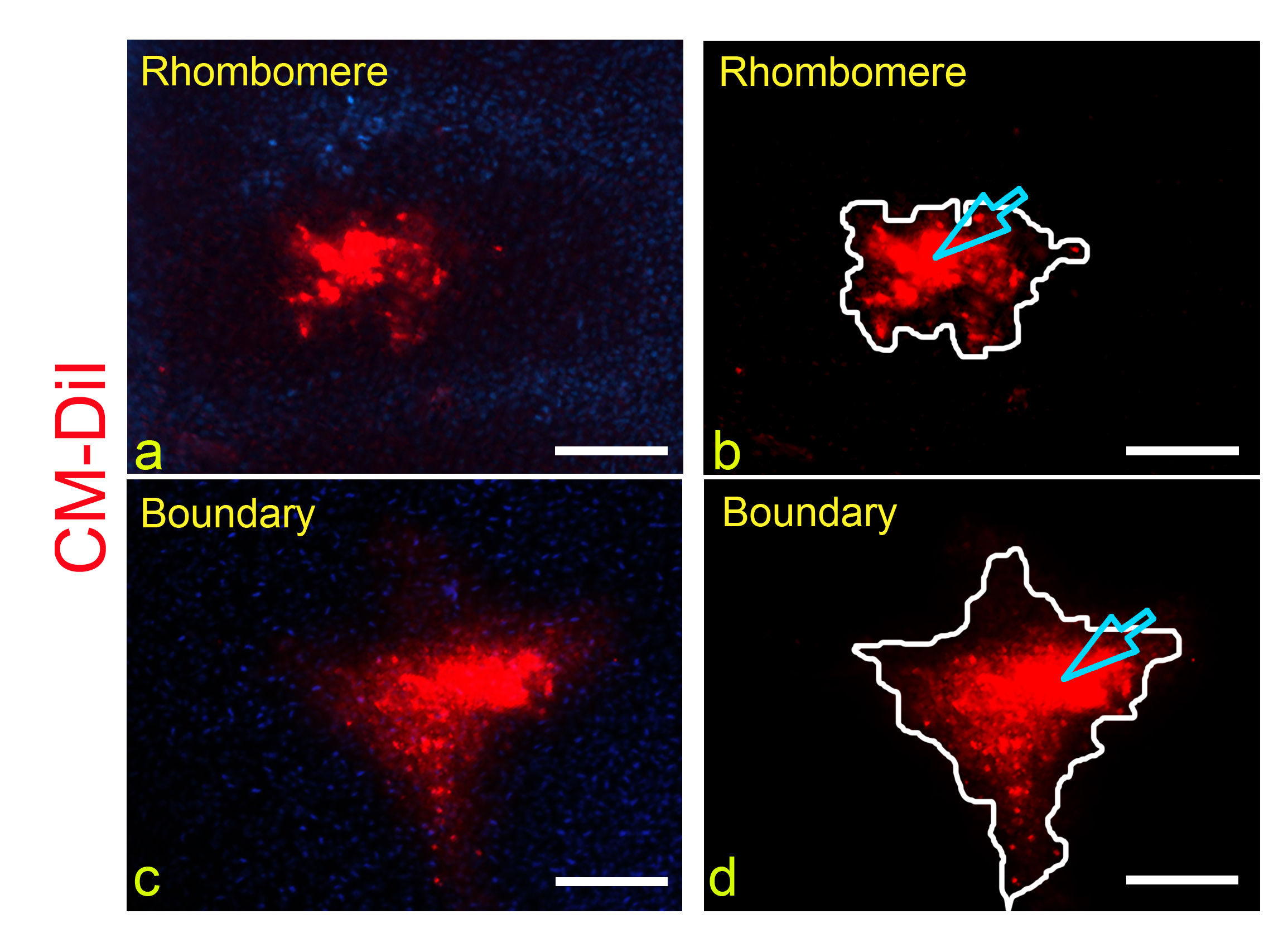

Supplement: Additional file 6: — CM-DiI labeling of boundary and rhombomere cells. Representative flat-mount confocal views of CM-DiI labelled rhombomere (a,b) or boundary (c,d) (n = 5 hindbrains). Arrows indicate injection site, yellow lines indicate boundaries. Outlined areas in (b,d) show dye expansion. (TIF 11336 kb) [file 12915_2016_277_MOESM6_ESM.tif]

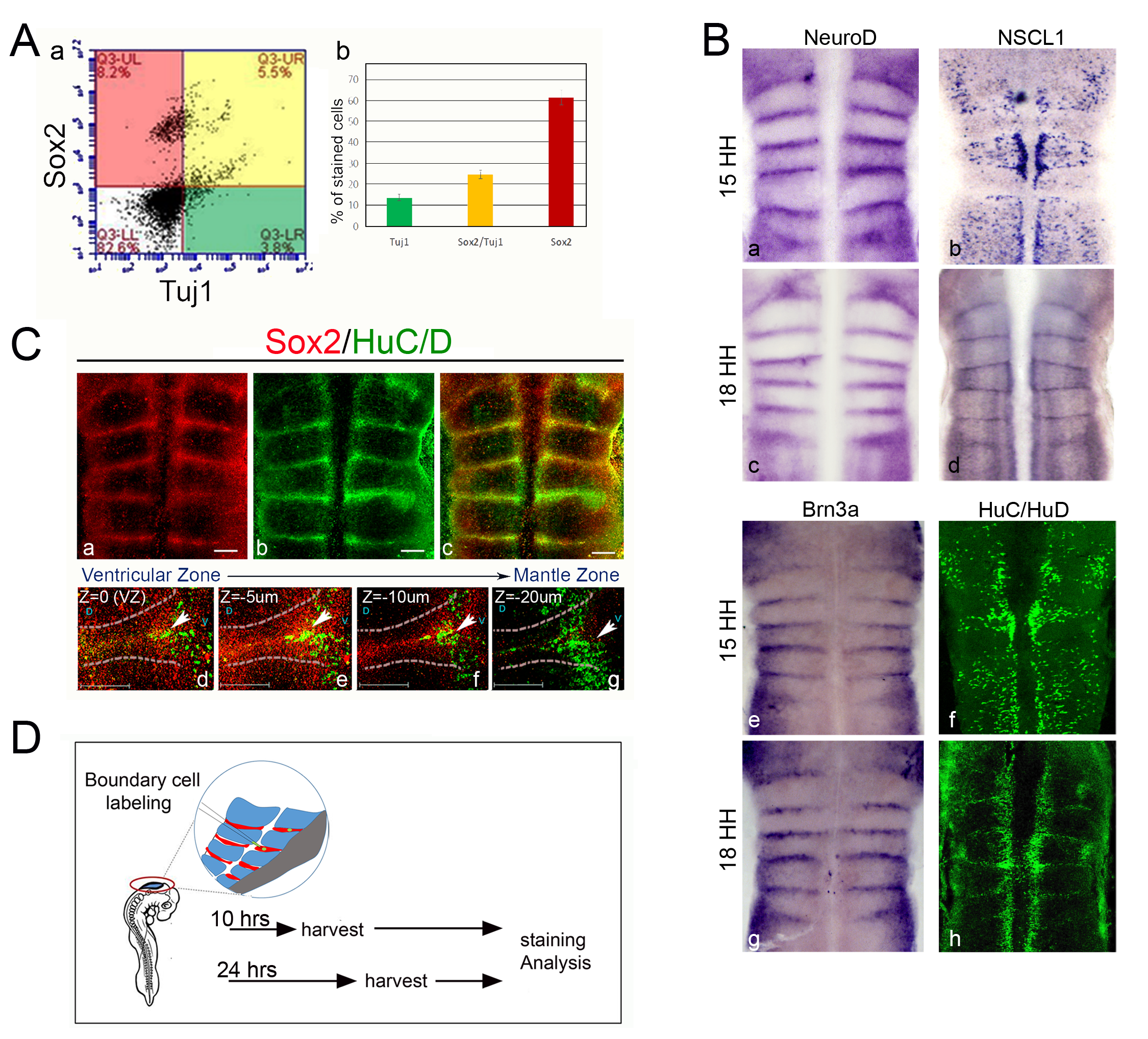

Supplement: Additional file 7: — Expression of neural-differentiation markers in the hindbrain. A. (a) Representative flow cytometry plot from 18 HH hindbrain stained with Sox2 and Tuj1. Quantification of relative abundance of Sox2/Tuj1-expressing cells is shown. (b) Graphic representation of Sox2/Tuj1 distribution as percentage of total stained cells. B. Representative flat-mounted views of 15HH and 18HH hindbrains in situ hybridized with RNA probes against NeuroD, NSCL1 and Brn3a, or immunostained for HuC/D (n = 10/marker) (e-h). Expression of NSCL1 and HuC/D shifts from punctuated rhombomeric expression in 15HH to boundary-enhanced expression at 18HH. C. (a-c) Representative flat-mounted views of 18HH hindbrains stained for Sox2 and HuC/D (n = 10). Merged image is shown in (c). (d-g) Sequential Z-stack analysis from 0 to –20 μm of a boundary area. Arrows indicate site of neural differentiation. Scale bars = 100 μm. D. Scheme of the clonal-analysis of HB cell-labeling experiment using injection of AFP plasmid into single cells and harvesting at two time points. (TIF 4496 kb) [file 12915_2016_277_MOESM7_ESM.tif]

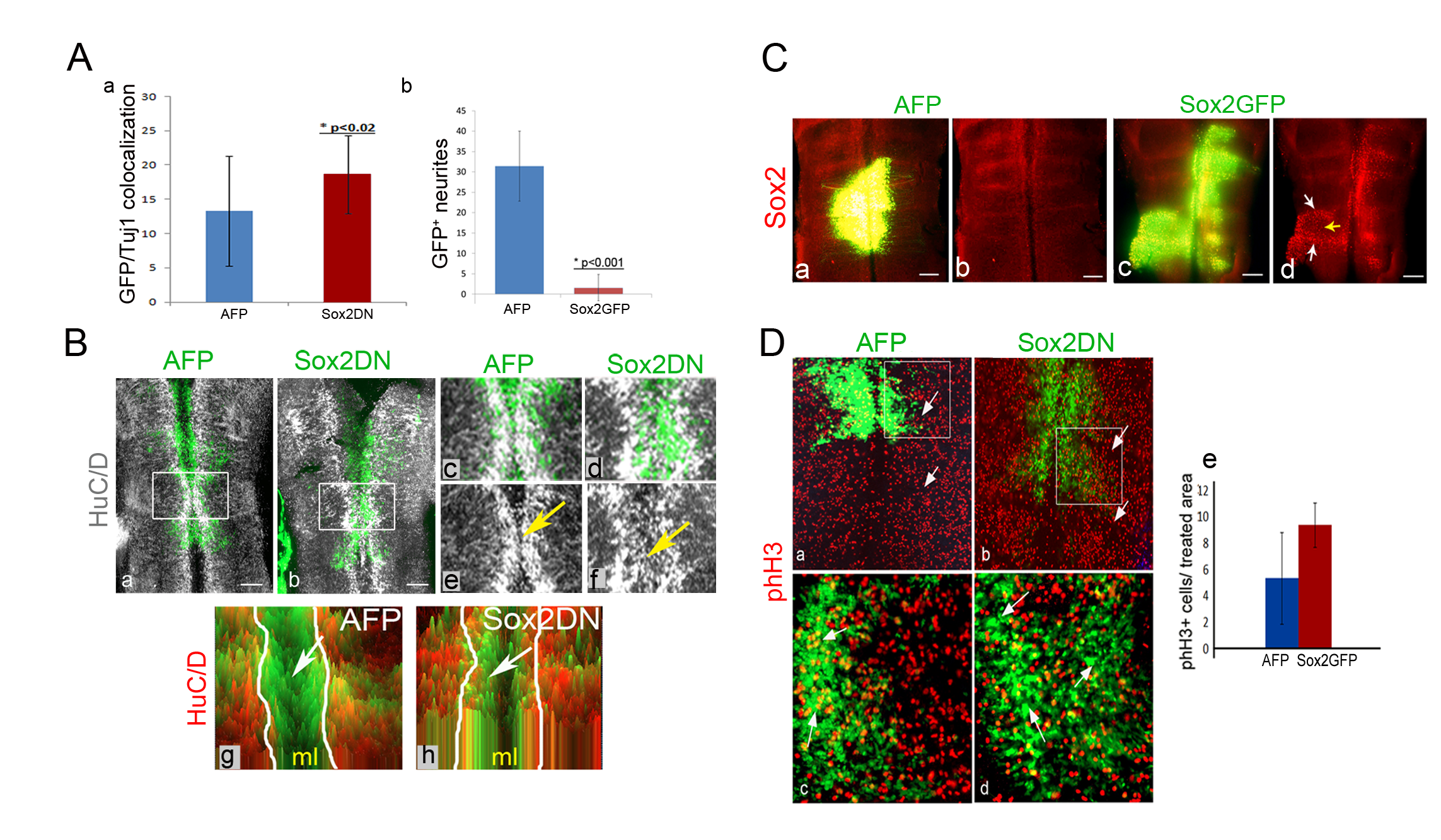

Supplement: Additional file 8: — Effects of Sox2 manipulation on hindbrain neural differentiation and cell proliferation. A. (a) Quantification of co-localization of Tuj1 with Sox2DN or AFP-expressing cells in primary hindbrain cultures (n = 11 hb/treatment). (b) Quantification of neurites expressing AFP (n = 14) or Sox2GFP (n = 12) plasmids in primary hindbrain cultures. B. (a-f) Representative flat-mounts views of 18HH hindbrains electroporated with AFP/Sox2DN (green) and stained for HuC/D (grey) (n = 5/treatment). High magnification views of boxed areas in (a,b) are shown in (c,e) for AFP and in (d,f) for Sox2DN. Yellow arrows indicate aberrant HuC/D expression. (g,h) 2.5D plots obtained from confocal analysis of flat-mounted hindbrains. White arrows indicate the midline. C. Representative flat-mounts of 18HH hindbrains electroporated with AFP (a,b) or Sox2GFP plasmids (green) and stained for Sox2 (red; n = 10). White arrows indicate areas of high Sox2 expression in electroporated HBs, yellow arrow indicates weaker upregulation of Sox2 in electroporated rhombomere. D. (a–d) Representative flat-mounts of 18HH hindbrains electroporated with AFP/Sox2DN plasmids (green) and stained for phH3 (red) (n = 5/treatment). Higher magnifications of boxed areas in (a,b) is shown in (c,d). Arrows in (a,b) indicate boundary regions. Arrows in (c,d) indicate electroporated cells with or without phH3, respectively. (e) Quantification by cell-counting of phH3+ cells per area in hindbrains electroporated with AFP or Sox2GFP plasmids (n = 11 hb/treatment). ml = midline. Scale bars = 100 μm. (TIF 2775 kb) [file 12915_2016_277_MOESM8_ESM.tif]
