# Supplementary material for: A new role of hindbrain boundaries as pools of neural stem/progenitor cells regulated by Sox2
Source: BMC Biol. 2016 Jul 8;14:57. doi: 10.1186/s12915-016-0277-y (PMC4938926; doi:10.1186/s12915-016-0277-y)
Supplement: Additional file 9: — Detailed description of clones and plasmids used in this study. (DOCX 13 kb) [file 12915_2016_277_MOESM9_ESM.docx]

**Plasmids and clones in use:**

**For *in-situ* hybridization:**

**NeuroD1**:

Reagent was received from D. Schultea. Plasmid backbone is pBSk. 5’ segment of NeuroD (1140bp) was cloned between sites ECoRV and Not1. Probe was prepared by *in-vitro* transcription of NeuroD1 antisense from pT3 of plasmid backbone.

**NSCL1:**

Chick EST clone 474F24 was cloned into pBS acquired from BBSRC, UK. Probe was prepared by *in-vitro* transcription of NSCL1 antisense from pT3 of plasmid backbone.

**Brn3A:**

Brn3A segment (accession # X91997) was cloned into pMos backbone vector, kindly provided by A. Graham. Probe was prepared by *in-vitro* transcription of Brn3A antisense from pT7 of plasmid backbone.

**For *in-ovo* electroporation:**

**Sox2 over-expression:**

The plasmid pMES-Sox2-IRES-GFP was kindly provided by D. Wu. In this plasmid the Sox2 full length cDNA was cloned in pMes backbone between xba1 and EcoRI downstream to chick pBactin promoter. IRES-GFP fragment was cloned downstream to the Sox2 sequence.

**pCMV/SV1-cSox2HMG-Engrailed (Sox2DN):**

Dominant negative form of Sox2 was kindly provided by Y. Kamachi and H. Kondoh. Vector consists of 400nt segment of Sox2 HMG box fused to Engrailed repressor domain (egr1), cloned into pCMV-SV1 expression vector backbone.
